# The DenA/DEN1 Interacting Phosphatase DipA Controls Septa Positioning and Phosphorylation-Dependent Stability of Cytoplasmatic DenA/DEN1 during Fungal Development

**DOI:** 10.1371/journal.pgen.1005949

**Published:** 2016-03-24

**Authors:** Josua Schinke, Miriam Kolog Gulko, Martin Christmann, Oliver Valerius, Sina Kristin Stumpf, Margarita Stirz, Gerhard H. Braus

**Affiliations:** Department of Molecular Microbiology and Genetics, Göttingen Center for Molecular Biosciences (GZMB), and Georg-August-University, Göttingen, Germany; University College Dublin, IRELAND

## Abstract

DenA/DEN1 and the COP9 signalosome (CSN) represent two deneddylases which remove the ubiquitin-like Nedd8 from modified target proteins and are required for distinct fungal developmental programmes. The cellular DenA/DEN1 population is divided into a nuclear and a cytoplasmatic subpopulation which is especially enriched at septa. DenA/DEN1 stability control mechanisms are different for the two cellular subpopulations and depend on different physical interacting proteins and the C-terminal DenA/DEN1 phosphorylation pattern. Nuclear DenA/DEN1 is destabilized during fungal development by five of the eight CSN subunits which target nuclear DenA/DEN1 for degradation. DenA/DEN1 becomes stabilized as a phosphoprotein at S243/S245 during vegetative growth, which is necessary to support further asexual development. After the initial phase of development, the newly identified cytoplasmatic DenA/DEN1 interacting phosphatase DipA and an additional developmental specific C-terminal phosphorylation site at serine S253 destabilize DenA/DEN1. Outside of the nucleus, DipA is co-transported with DenA/DEN1 in the cytoplasm between septa and nuclei. Deletion of *dipA* resulted in increased DenA/DEN1 stability in a strain which is unresponsive to illumination. The mutant strain is dysregulated in cytokinesis and impaired in asexual development. Our results suggest a dual phosphorylation-dependent DenA/DEN1 stability control with stabilizing and destabilizing modifications and physical interaction partner proteins which function as control points in the nucleus and the cytoplasm.

## Author Summary

Posttranslational modifications of protein can affect stability, activity, interaction and localization of targeted substrates. The covalent attachment of the small ubiquitin-like protein Nedd8 regulates the activity of E3 cullin-RING ligases and thereby the ubiquitin-mediated protein degradation via the ubiquitin-proteasome system. The two deneddylases COP9 signalsome (CSN) and deneddylase 1 (DenA/DEN1) reverse the process of neddylation. Increased deneddylase activity is associated with human cancer. Both deneddylases physically interact with each other, which is conserved from fungi to human, and have different substrate affinities resulting in the regulation of distinct developmental programs in the filamentous fungus *Aspergillus nidulans*. We found in this study that DenA/DEN1 stability depends on its phosphorylation status and on its cellular localization in either the nucleus or the cytoplasm during fungal development. Cytoplasmatic stability control of DenA/DEN1 depends on a novel DenA/DEN1 interacting phosphatase, DipA, which is involved in septa formation required for cellular compartmentalization and fungal differentiation. These findings shed light on the complex interplay between the two deneddylases during development and open up new perspectives for future studies concerning cell division, differentiation and might be even relevant for a better understanding of tumor formation.

## Introduction

The attachment of chemical groups or proteins to substrate amino acid residues extends the functional diversity and dynamics of the cellular proteome. These posttranslational modifications (PTMs) change protein surfaces and lead to differences in the interaction with other proteins thereby affecting stability, activity and localization of targeted substrates [1–4]. PTMs such as phosphorylation, ubiquitination and neddylation are involved in a wide range of cellular processes including development, cell-cycle control, DNA repair, cell growth and signaling [2,3]. Phosphorylation can serve as a signal that triggers subsequent ubiquitination of the modified substrate [5–7]. Ubiquitination is the covalent binding of the polypeptide ubiquitin to proteins and serves predominantly as a signal for degrading the labelled protein via the 26S proteasome [8–10]. The transfer of ubiquitin onto a substrate is mediated by E3 ubiquitin ligases. The largest group within E3 ubiquitin ligases is the cullin-RING E3 ubiquitin ligase (CRL) family [11]. CRLs are multi-protein complexes with a central cullin which serves as a scaffold to mediate substrate binding and ubiquitin transfer. Neddylation is a process highly related to ubiquitination. The covalent binding of the ubiquitin-like (ubl) protein Nedd8 activates CRLs. Neddylation of cullins leads to a conformational change that facilitates the transfer of ubiquitin to a substrate resulting in its degradation [12]. Dynamic exchanges of the substrate adaptor are regulated by CAND1 which promotes the assembly of new F-Box containing cullin-RING ligases and controls the recruitment of less abundant substrates [6,13–15].

Most PTMs are reversible, thus not only the modification itself, but also the removal of PTMs plays an important role to understand the impact on affected pathways. Ubiquitination and neddylation are reversed by deubiquitinases and deneddylases, respectively. Two deneddylases are described in eukaryotes, the COP9 signalosome (CSN) and the deneddylase1 (DEN1) corresponding to DenA in *Aspergillus nidulans* [16]. CSN was initially discovered in *Arabidopsis thaliana* as a suppressor of light-dependent development [17]. It is conserved from fungi to human and consists of up to eight subunits (CSN1-CSN8). The CSN complex shares similarities regarding structure and subunit composition with the LID of the 26S proteasome and with the eukaryotic translation initiation factor 3 (eIF3). Each of them possesses six subunits with a PCI (proteasome-LID, CSN, eIF3) and two subunits with an MPN (Mpr1p and Pad1p N-terminal) domain [18–20].

The six PCI domain proteins of human CSN form a horseshoe-like ring and all eight subunits are connected by a bundle of C-terminal α-helices [21]. The only intrinsic enzymatic function of the CSN complex is represented by its MPN isopeptidase activity against cullins modified with Nedd8 [22,23]. Its deneddylase activity is harbored within CSN5 which is the last subunit that is integrated into a seven subunit pre-complex [24,25]. All eight CSN subunits are required for the holocomplex acting as denedddylase. Loss of one subunit leads to impairment of the entire active complex causing accumulation of neddylated substrates [25–28]. Through the regulatory function of CSN in ubiquitin-dependent protein degradation it is involved in complex cellular processes such as DNA repair and cell development [29–33]. Beside its primary function as deneddylase, CSN acts as an assembly platform recruiting a variety of proteins involved in protein modification such as ubiquitination and phosphorylation [34–37].

The second known deneddylase DenA/DEN1 represents another CSN binding protein which physically interact in the nucleus. This interaction is conserved from fungi to human and results in degradation of DenA/DEN1 [16]. The interaction of DenA/DEN1 with CSN has evolved in different organisms from CSN2 in *Schizosaccharomyces pombe* to CsnG/CSN7 in *A*. *nidulans* or CSN1 in human cells as distinct major interacting CSN subunits [16]. Both deneddylases act towards Nedd8 conjugates but they differ in their substrate specificity and subcellular distribution. Compared to CSN, whose main substrates are neddylated cullins, DenA/DEN1 has a higher affinity towards non-cullin proteins [16,38,39]. However, it can act towards neddylated cullins *in vitro* in a concentration-dependent manner [40,41]. Low concentrated human DenA/DEN1 deconjugates polyneddylated cullins to yield a mononeddylated form. Elevated concentrations of DenA/DEN1 are capable to catalyze the complete removal of Nedd8 from cullins. In contrast, human CSN did not efficiently cleave polyneddylated cullins, suggesting that they are not the main substrate [40]. CSN has been predominantly described to be localized in the nucleus of plant and human cells [33,42,43], whereas the cellular localization of fungal DenA/DEN1 includes a nuclear as well as a dominant cytoplasmatic subpopulation located mainly at septa [16]. The septa are internal cross walls between fungal cellular compartments connected by pores.

Both deneddylases are involved in different developmental programs in the filamentous ascomycete *A*. *nidulans*. Fungal vegetative growth in liquid culture results in branched filaments divided by septa. Vegetative hyphae can differentiate either sexually or asexually, depending on the environmental conditions [44–46]. The fungal CSN complex prevents the accumulation of development specific E3 ubiquitin cullin-RING ligases and is required for sexual development [25,47,48]. Gene deletion of DenA/DEN1 has no obvious consequences in a variety of eukaryotic organisms, but revealed in *A*. *nidulans* that DenA/DEN1 contributes to asexual spore formation during limited pyrimidine supply [16]. *A*. *nidulans* possesses the full set of eight CSN subunits (CsnA-CsnH), including the catalytic active subunit CsnE. This subunit composition corresponds to the situation found in plants and humans [25,42]. Other fungi such as yeast or *Neurospora crassa* express only smaller complexes where some genes for subunits are not present in the corresponding genomes [49,50]. *A*. *nidulans* represents an attractive model to study the function and interrelation of CSN and DenA in multicellular development, due to the distinct impact of the two deneddylases on individual fungal developmental programs.

The focus of this study was the stability control and the functions of the nuclear and cytoplasmatic subpopulations of the deneddylase DenA using the multicellular mold *A*. *nidulans* as a model organism. It was found that five of the eight CSN subunits destabilize the nuclear DenA subpopulation, which has an auxiliary role in deneddylating cullins in the absence of a functional CSN. DenA is phosphorylated and a change of its phosphorylation pattern from vegetative growth to asexual development induces its degradation. The DenA interacting phosphatase DipA was identified as binding partner which targets cytoplasmatic DenA for degradation. This fungal specific phosphatase is co-transported with DenA to septa and nuclei. The gene for DipA is essential for septa positioning and light regulated fungal development.

## Results

### Fungal DenA provides an auxiliary function to support deneddylation and development in the absence of the seventh CSN subunit CsnG

Deletion mutant strains with defects in DenA/DEN1 or CSN encoding genes accumulate different subsets of neddylated proteins [16,25]. This suggests that the substrate specifity for both deneddylases is not identical which was studied in more detail. Western hybridization with a *denA/csnE* double deletion strain defective in both deneddylases resulted in the accumulation of cullins with higher molecular mass which had been described as hyperneddylated cullins (S1 Fig), [16]. As these cullin variants were absent in the deneddylase single deletion strains and appeared only in the *denA/csnE* double deletion strain, both fungal deneddylases are presumably able to process hyperneddylated cullins to yield a mononeddylated variant.

**Fig 1:**
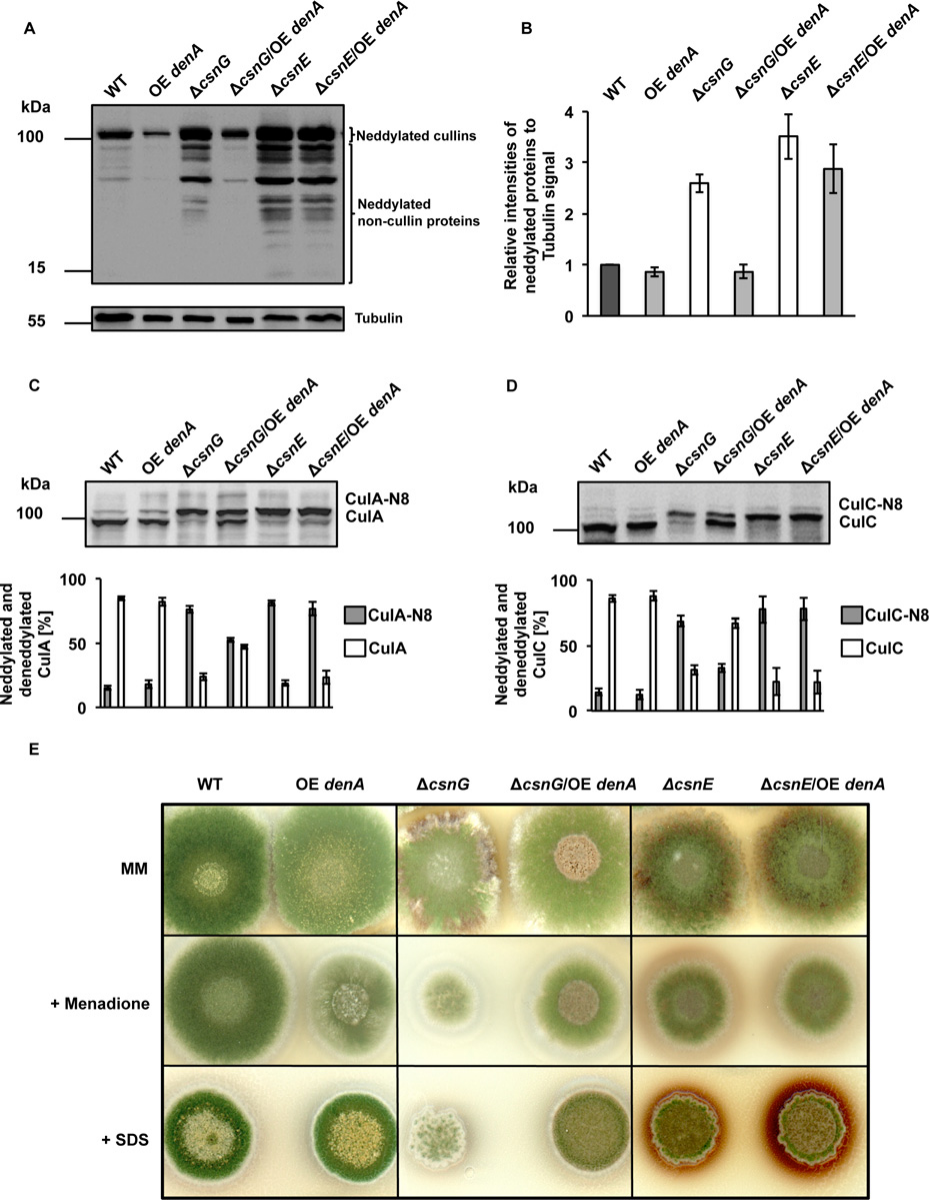
Changes in deneddylase activity and its consequences for the cellular pool of neddylated proteins and fungal development. **(A)** Western analyses with Nedd8 or Tubulin (reprobed as loading control, lower part) antibodies of vegetative grown mycelia of *A*. *nidulans*. Wild type (WT) was compared to mutant strains with altered deneddylase activity either with decreased COP9 signalosome (Δ*csnG*, Δ*csnE*) or increased *denA* (OE *denA*) and combinations of defective CSN and increased DenA (Δ*csnG*/OE *denA*, or Δ*csnE*/OE *denA*). Neddylated cullins correspond to ≈ 100 kDa and faster migrating bands are summarized as neddylated non-cullin proteins. **(B)** Semi-quantitative analyses of Nedd8 signal intensities of three independent experiments of strains shown in (A) normalized to Tubulin signals, including standard deviations. **(C)** Western hybridization using cullinA (CulA) or **(D)** cullinC (CulC) antibodies and determination of the ratios (lower panels) of neddylated (CulA-N8 or CulC-N8) in comparison to deneddylated cullins of three independent experiments. **(E)** Cellular deneddylase activity and fungal development. Equal amount of spores of indicated strains were point-inoculated and grown for four days under illumination which induces asexual development in wild type. Respective strains are shown either on minimal (MM) or stress inducing media (+menadione, +SDS).

Overlapping substrate specificity of the two deneddylases was further investigated by analyzing whether increased amount of DenA can compensate the accumulation of neddylated proteins in *csn* deficient strains. The effect of overexpressed *denA* on cellular neddylation levels was analyzed in a *csnG* deletion strain, which lacks the major DenA interaction partner, and in a *csnE* deletion strain, defective in the catalytic deneddylase subunit. Both mutants do not contain an active CSN holocomplex. A *denA* overexpression construct was ectopically integrated into wild type, *csnG* and *csnE* mutant strains. Protein crude extracts of vegetative grown wild type, overexpression *denA* (OE *denA*), Δ*csnG*, Δ*csnG*/OE *denA*, Δ*csnE* and Δ*csnE*/OE *denA* strains were compared by western hybridization using Nedd8 antibody (Fig 1A and 1B). In strains deleted for *csnG* or *csnE* the intensity of neddylated proteins, including cullins as well as lower weight non-cullin proteins, was 2.5 fold of the value detected for wild type or OE *denA* strains. Whereas increased *denA* expression in *csnE* deficient strain did not affect the neddylation pattern, overexpression of *denA* in Δ*csnG* strain reduced neddylated proteins, meaning that the overall neddylation profile of Δ*csnG*/OE *denA* strain was similar to the pattern that was detected in wild type.

The effects of high DenA amounts on neddylation levels of CSN specific substrates such as cullin-1 (CulA) and cullin-3 (CulC) were compared (Fig 1C and 1D). Most cullins in wild type cells were unconjugated and less than 20% of the total cellular CulA and CulC pools were neddylated. Overexpression of *denA* did not affect the ratio of neddylated versus unneddylated cullins in wild type. There were distinct effects when *denA* was overexpressed in different *csn* mutant strains. Deletion of either *csnG* or *csnE* resulted in a drastic increase in neddylated cullins, where 70% of the cullins were neddylated and only 30% were unneddylated. Overexpression of *denA* had no influence on cullin neddylation levels in a Δ*csnE* strain (Δ*csnE*/OE *denA*). In contrast, high levels of DenA in a *csnG* deletion strain (Δ*csnG*/OE *denA*) changed the cullin neddylation pattern towards a higher ratio of unneddylated cullins (50% for CulA or 70% for CulC).

*A*. *nidulans* strains deleted for CSN encoding genes are viable and display pleiotropic phenotypes which is different from the embryonic lethality observed in mammals and plants [25,42,51]. It was shown that deletion of *csnG* or *csnE* results in a block of fungal sexual development, reduced asexual spore production, and impaired secondary metabolism [25,47]. The latter has an increasing importance for fungal pathogenicity and is indicated by the accumulation of reddish orcinol derivatives within hyphae and the surrounding medium [25,32,52]. It was analyzed whether high levels of DenA affect the developmental phenotypes of a *csnG* deletion strain. Overexpression of *denA* had neither an effect on asexual development in wild type background nor in a *csnG* or *csnE* deletion strain during cultivation on minimal medium (Fig 1E, upper panel). When grown in the presence of stress inducing agents such as menadione, causing oxidative stress, or SDS, causing protein damage, the Δ*csnG*/OE *denA* strain revealed a colony that appeared healthier in terms of size and green pigmentation when compared to *csnG* mutant (Fig 1E, lower panels).

These results imply that DenA can partially compensate the lack of CsnG but not of CsnE, suggesting that one of these CSN subunits has an additional function on DenA, which the other subunit does not have.

### Three PCI CSN subunits as well as both MPN subunits destabilize DenA in the nucleus

**Fig 2:**
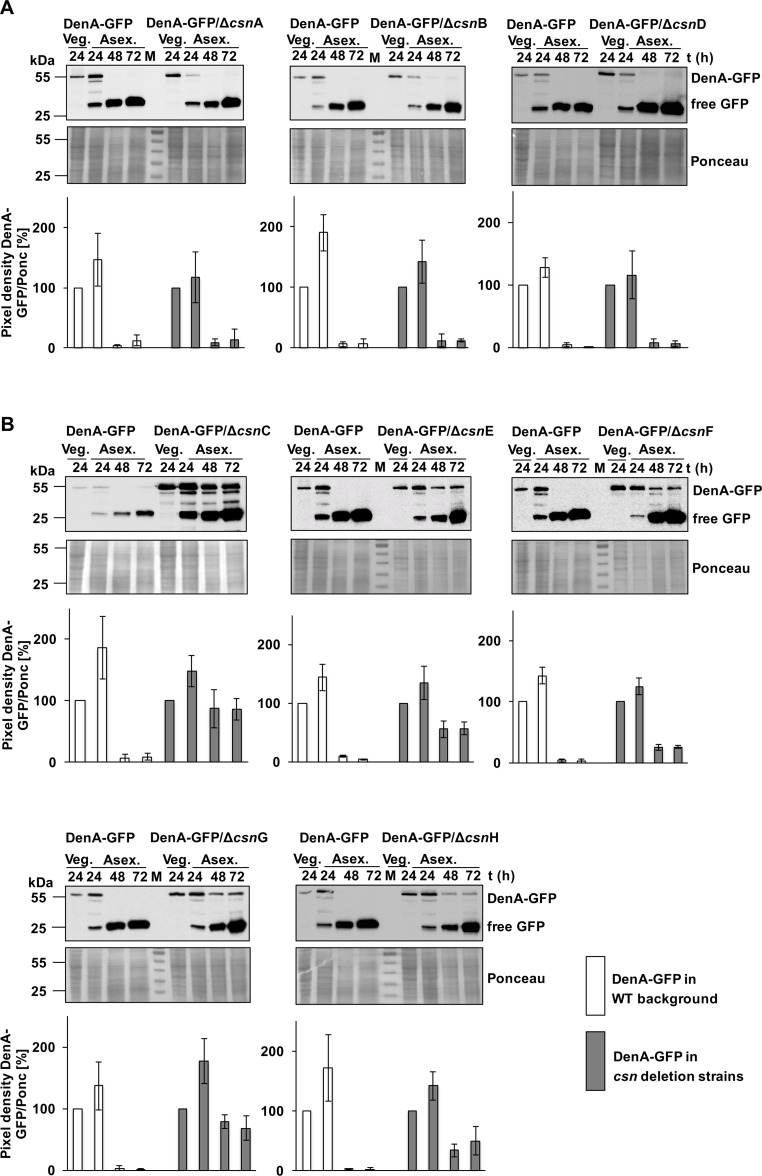
DenA protein levels during fungal development. DenA is stable during vegetative growth but not during illumination corresponding to advanced wild type asexual development. **(A)** Protein level of DenA-GFP (54.5 kDa) is monitored in presence (*csnA*, *csnB*, *csnD*) or absence (Δ*csnA*, Δ*csnB*, Δ*csnD*) of indicated CSN subunit encoding genes by western hybridization with GFP-antibody. Protein extracts from indicated time points (in hours) of vegetative (Veg.) or illumination induced development (Asex.) were applied. The signal of free GFP (25 kDa) corresponds to DenA degradation. Loading control: Ponceau staining (below). Lower panels show quantification of band intensities of DenA-GFP relative to vegetative growth. **(B)** Western hybridization with GFP-antibody of DenA-GFP in wild type in comparison to Δ*csnC*, Δ*csnE*, Δ*csnF*, Δ*csnG* or Δ*csnH* strains. DenA is present throughout development in respective deletion strains. Quantifications of DenA-GFP protein levels during development relative to vegetative growth are shown in the lower panels.

CsnG was reported to be a direct physical binding partner of nuclear DenA resulting in DenA degradation during asexual development inducing conditions [16]. It is unknown whether CsnG is the only COP9 signalosome subunit causing destabilization of DenA. To investigate the impact of all eight fungal CSN subunits on DenA stability, *denA* was replaced at its original locus by a *denA-gfp* fusion driven by its own promoter and combinations of this strain with all eight single *csn* deletion backgrounds (Δ*csnA* to Δ*csnH*) were constructed. DenA-GFP protein amounts were visualized by GFP antibodies in western hybridizations during vegetative growth and at different time points of asexual development. The DenA-GFP fusion product was expressed and stable during vegetative growth in wild type as well as in *csnA*, *csnB* or *csnD* deletion backgrounds. When asexual conditions were induced by illumination DenA-GFP was degraded and no more visible after 48h or 72h (Fig 2A). Free GFP, which is a stable remnant from degradation of the DenA fusion protein, accumulated during development in all tested strains. Deletion of *csnC*, *csnE*, *csnF*, *csnG* or *csnH* resulted in a different effect on DenA stability. In all these strains DenA-GFP was still detectable even after 72h, suggesting DenA-GFP is present during the entire illumination period inducing asexual development in wild type (Fig 2B). This effect was most pronounced in the *csnC* deletion strain, where 86 ± 17% of DenA-GFP was still visible after 72 h of asexual development compared to vegetative growth.

These data suggest that DenA is stable during vegetative growth and converts into an unstable protein after the induction of asexual development. Degradation of DenA requires the five intact subunits CsnC, E, F, G and H which are arranged side by side forming a joint surface according to the solved structure of human CSN [21]. The entire COP9 signalosome is not required for this developmental control of DenA stability, because the subunits CsnA, B or D are dispensable for the DenA turnover control.

### The transition from stable vegetative DenA to an unstable protein during development coincides with an additional C-terminal serine phosphorylation at S253

**Fig 3:**
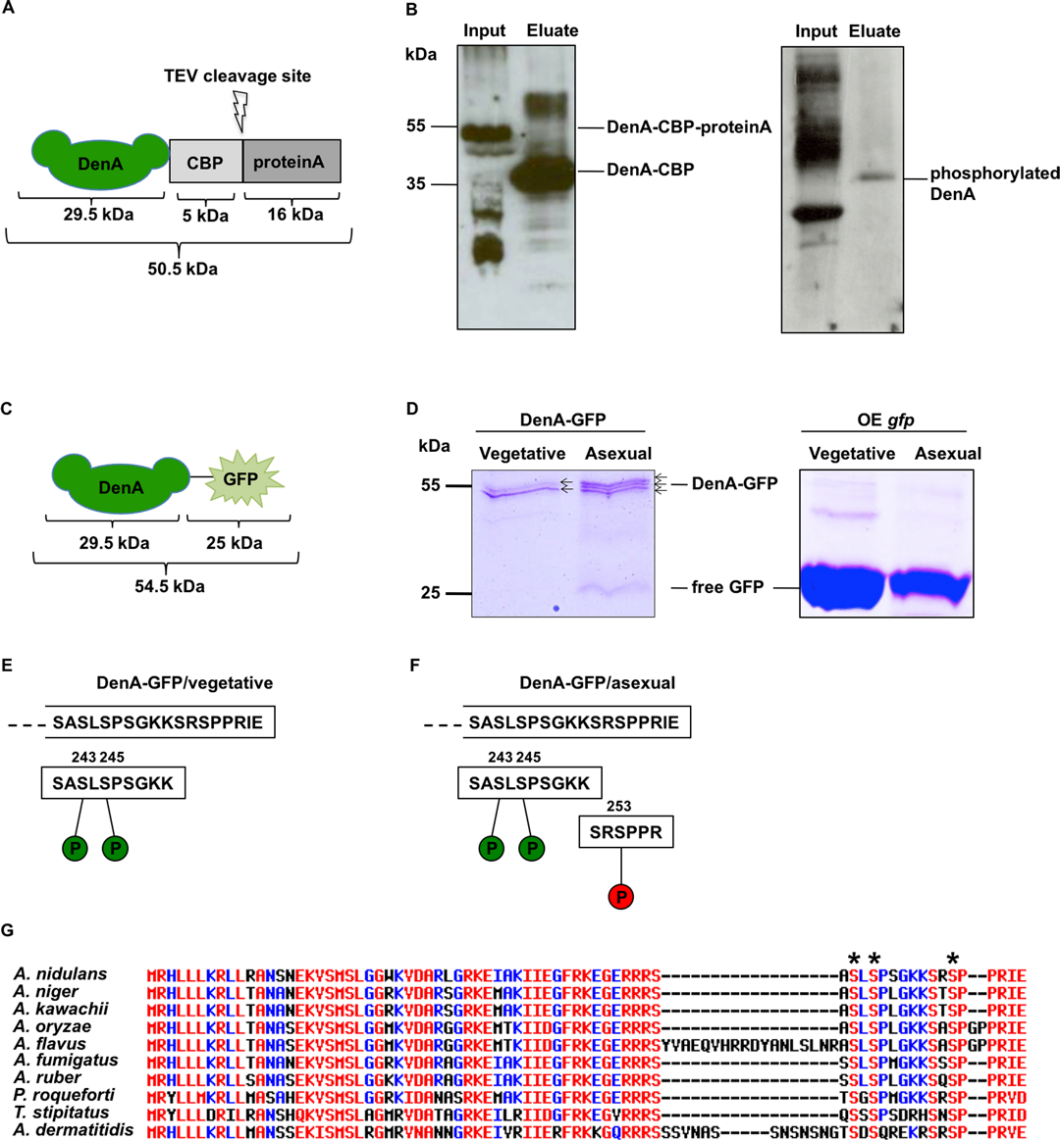
DenA phosphorylation variants. **(A)** C-terminally tagged DenA-CBP-proteinA (50.5 kDa) used for TAP purification. **(B)** Enriched vegetative DenA-CBP (34.5 kDa) after TEV cleavage and elution compared to the input, visualized by western hybridization with calmodulin-binding protein antibody (CBP) (left column). Phosphorylated DenA was detectable after reprobing the membrane with ser/thr phosphorylation specific antibody (right column). **(C)** C-terminally tagged DenA-GFP (54.5 kDa) used for GFP affinity purification. **(D)** Colloidal blue stained Phos-tag gel with DenA-GFP extracted from 24h vegetative and 24h illuminated grown cultures (left column). Arrows indicate the number of different DenA isoforms. Control: overexpressing GFP (OE *gfp*) strain (right column). **(E)** Deduced amino acid sequence of DenA C-terminus. LC-MS/MS analyses identified one peptide with two phosphorylated serines at position S243 and S245 of DenA during vegetative growth and **(F)** two peptides with three phosphorylated serines at positions S243, S245 and S253 during asexual development. **(G)** Multiple alignments of DenA C-termini of *Aspergillus nidulans*, *Aspergillus niger*, *Aspergillus kawachii*, *Aspergillus oryzae*, *Aspergillus flavus*, *Aspergillus fumigatus Z5*, *Aspergillus ruber*, *Penicillium roqueforti*, *Talaromyces stipitatus* and *Ajellomyces dermatitidis*. Identified phosphorylation sites are marked with asterisks. Red: high (90%), blue: low (50%) consensus values [56].

Posttranslational modifications of protein can affect activity, localization and stability of respective substrates [3]. As DenA was stable during vegetative growth but became unstable during development, it was studied whether DenA degradation is linked to changes in posttranslational modifications. Previous studies demonstrated that CSN associated kinases can phosphorylate and destabilize target proteins interacting with the COP9 signalosome [53,54]. Consequently, DenA phosphorylation was analyzed during vegetative growth and during the course of asexual development. DenA-CBP-proteinA (DenA C-terminally tagged with calmodulin-binding protein and proteinA) was expressed from the respective fusion gene under control of the native *denA* promotor that was integrated at the *denA* locus. The resulting fusion protein (Fig 3A) was enriched from vegetative cultures by tandem-affinity purification (TAP). The presence of DenA-CBP was monitored by western hybridization using CBP-antibody. Treatment of the membrane with serine/threonine phosphorylation specific antibody generated a signal at the expected mass of DenA-CBP indicating that DenA is phosphorylated (Fig 3B). The fused TAP-tag was replaced by GFP, which enables a more efficient purification method. A *denA-gfp* fusion construct driven by its native promoter at the original locus was used to enrich DenA-GFP by GFP-trap (Fig 3C). DenA-GFP was extracted from 24h vegetative grown and 24h asexually developed cells for comprehensive insights about DenA phosphorylation during the transition from vegetative growth to asexual development. A strain overexpressing ectopically integrated GFP (OE *gfp*) served as control. SDS gels and western hybridization applying GFP antibodies were used to verify the enrichment of DenA-GFP and OE GFP (S2 Fig). Phosphorylation variants of enriched DenA-GFP were examined by phos-tag acrylamide gels (Fig 3D). Enriched DenA-GFP displayed different mobility shifts suggesting distinct phosphorylation variants of DenA-GFP in vegetative growth and asexual development. The control strain revealed that overexpressed GFP was not modified by phosphorylation as only a single band was observed during vegetative growth and after the induction of development. Two vegetative in comparison to three asexual DenA-GFP versions indicated that there are developmental-specific changes in phosphorylation after the induction of asexual development. Differences in DenA phosphorylation pattern of vegetative and asexually extracted DenA-GFP were verified by LC-MS/MS and could be localized to three serine residues at DenA C-terminus (S3 Fig). Stable DenA isolated from vegetative cultures possessed phosphorylation sites at serine 243 and serine 245 (S243, S245). DenA enriched after induction of asexual development carried an additional phosphate residue at serine 253 (S253) (Fig 3E and 3F). Analyses of the three identified DenA phosphorylation sites using a computational prediction tool [55] suggested the cyclin dependent cell cycle kinase Cdc2 as a potential kinase responsible for phosphorylating DenA at the serines S245 and S253.

Multiple alignments of DenA C-termini from different species revealed a partial conservation of identified phosphorylation sites within the fungal kingdom. The modified serines are found in a wide range of fungi (*A*. *nidulans*, *A*. *ruber*, *Talaromyces stipitatus*) and are also present in industrially used (*A*. *niger*, *A*. *kawachii*, *A*. *oryzae*, *A*. *fumigatus Z5*, *Penicillium roqueforti*) and clinically relevant (*A*. *flavus*, *Ajellomyces dermatitidis*) fungal strains (Fig 3G, phosphorylated serines are marked with asterisks). They are absent in *Aspergilli* as *A*. *clavatus*, *A*. *fumigatus Af293* or *A*. *terreus* and are neither present in plants nor animals (S4 Fig).

These data demonstrate that DenA is phosphorylated at the C-terminal end during vegetative growth and development. Vegetative stable DenA possesses two phosphorylated residues at positions S243 and S245. The transition to an unstable DenA during differentiation correlates with the appearance of an additional developmental-specific phosphate group at serine S253 of the 258 amino acids long protein.

### Phosphorylation of DenA S253 destabilizes the protein during fungal development

**Fig 4:**
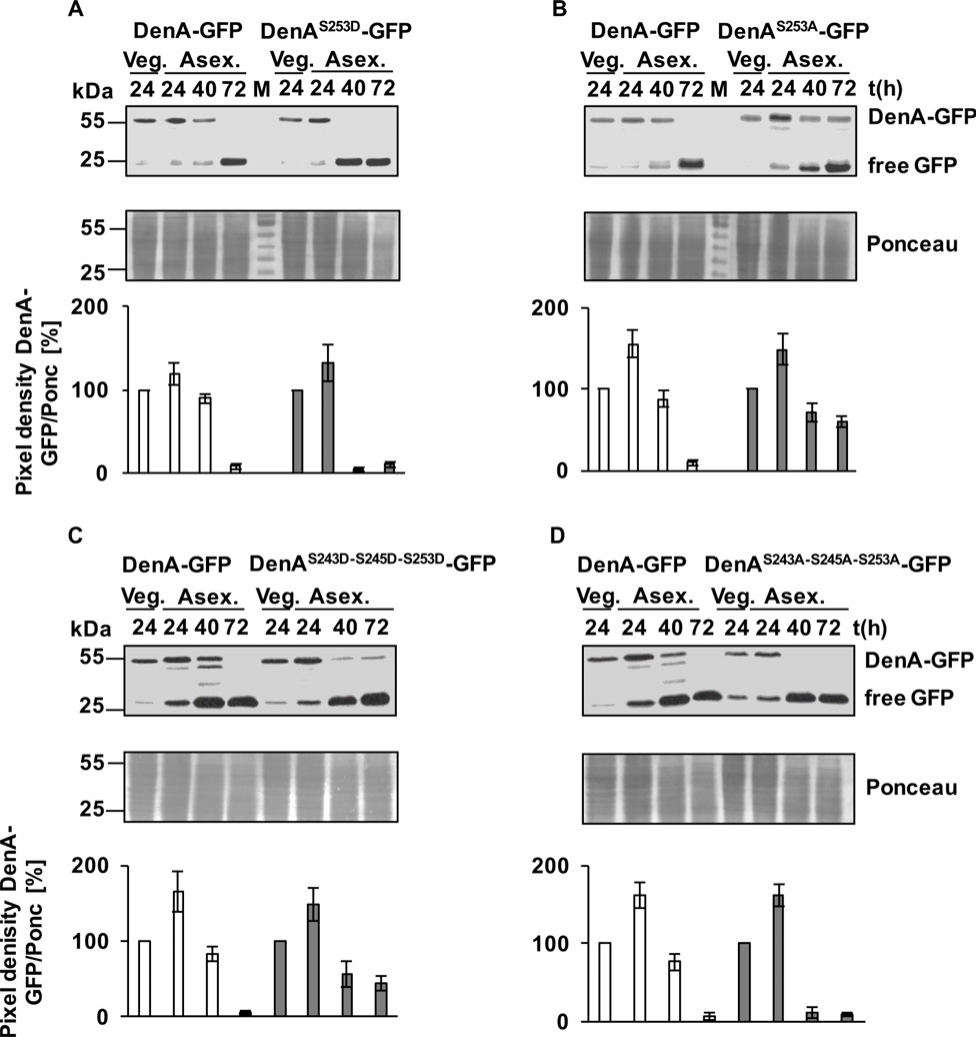
Protein amount of DenA-GFP and amino acid substituted variants during development. Western hybridization with equal amounts of protein extracts of DenA-GFP (54.5 kDa) compared to **(A)** DenA^S253D^-GFP carrying a negative charge reminiscent of a phosphorylated protein, **(B)** DenA^S253A^-GFP which cannot be phosphorylated, **(C)** DenA^S243D-S245D-S253D^-GFP with three negative charges mimicking a triple phosphorylated protein and **(D)** the corresponding DenA^S243A-S245A-S253A^-GFP which cannot be phosphorylated. Samples were taken from vegetative hyphae (Veg) and at indicated time points (in hours) of illumination which induces asexual development (Asex). Membranes were treated with GFP-antibody to visualize the fusion protein and free GFP (25 kDa). Loading control: Ponceau staining. Lower panels show quantification of band intensities of DenA-GFP and its respective variants relative to vegetative growth.

The role of the additional S253 phosphorylation site of DenA was analyzed to determine whether it is relevant for the destabilization of the DenA protein during asexual development. The S253 codon was either exchanged for the negative charged aspartic acid (DenA^S253D^) mimicking constant phosphorylation at this site or for neutral alanine (DenA^S253A^), which cannot be phosphorylated at this residue. These *denA* variants were fused C-terminally to GFP and integrated at the endogenous *denA* locus. Proteins from vegetative mycelium as well as under conditions inducing asexual development were extracted at various time points and used for western hybridization applying GFP antibody. Wild type DenA-GFP was present in vegetative growth, decreased during early stages of asexual development and was undetectable after 72h. The simulated phosphorylated DenA^S253D^ variant was unstable and already undetectable after 40h of asexual development, indicating a premature degradation of the protein (Fig 4A). In contrast, the amount of DenA^S253A^, mimicking a dephosphorylated protein at this site, was present throughout the entire asexual development suggesting that the protein is even more stable than wild type DenA which was undetectable after 72 hours of asexual development (Fig 4B). This suggests that DenA stability is controlled by phosphorylation during asexual development.

The next step was to address the function of S243 and S245 in the destabilization of DenA through the adjacent S253 phosphorylation site after the induction of asexual development. A DenA variant was constructed where all three phosphorylated serine codons (S243, S245, S253) were exchanged for aspartic acid (D) to introduce negative charges. The triple aspartic acid variant DenA^S243D-S245D-S253D^-GFP, mimicking a constant phosphorylated version at these positions, was less unstable compared to wild type as it was still detectable even 72 hours after the induction of asexual development (Fig 4C). This is in contrast to DenA wild type which stayed stable during initial asexual development but degraded after 72 hours and is also different from the DenA S253D variant which was already undetectable after 40 hours of asexual development. The triple aspartic acid version is reminiscent of the S253A variant which was also stabilized during development. These results suggest that S243 and S245 phosphorylation stabilize DenA within the fungal cell during development, whereas S253 phosphorylation has a destabilizing function. To analyze whether the stabilization function of S243/S245 of DenA can be verified all three serine codons were exchanged for alanine (A) codons representing unmodified residues. The triple alanine DenA^S243A-S245A-S253A^-GFP version, which cannot be phosphorylated at these positions, was as unstable as S253D variant and was undetectable after 40h of asexual development (Fig 4D).

An *A*. *nidulans* strain deleted for *denA* is impaired in asexual development during limited pyrimidine supply [16]. To determine whether changes in DenA stability affect fungal development the effect of premature DenA degradation on asexual development was analyzed. Both DenA triple mutants (DenA^S243A-S245A-S253A^ and DenA^S243D-S245D-S253D^) were C-terminally tagged and the respective fusion genes, driven by an inducible promoter, were ectopically integrated in the *denA* deletion strain. The prematurely destabilized DenA^S243A-S245A-S253A^ variant was unable to complement the *denA* deletion phenotype as it displayed the same reduction in asexual development during partial pyrimidine limitation as observed for the *denA* deletion strain (S5 Fig). In contrast, the stabilized DenA^S243D-S245D-S253D^ variant produced asexual spores similar to wild type.

This suggests that phosphorylation of DenA at residues S243 and S245 has a protective function for its protein stability, whereas phosphorylation of S253 promotes DenA degradation. Stable DenA is necessary for the initial phase (until 40 hours) of asexual development to allow the appropriate formation of mature fungal conidiospores.

### DenA co-purifies the DenA interacting phosphatase DipA

The complex interplay between stabilizing C-terminal serine residues and the destabilizing S253 of DenA suggests that a phosphatase is required for protein degradation of DenA during asexual development. Physically DenA interacting proteins were examined by co-purification experiments with DenA tagged either with TAP or GFP. Interacting candidates were extracted from vegetative protein extracts and identified by LC-MS/MS. Putative binding proteins were considered as reliable when they were found in both purification methods with at least two different peptides but were absent in the respective control strains. In total five identified proteins fulfilled these requirements and were considered as putative DenA interacting proteins (Dip, Table 1).

**Table 1:** DenA and DenA interacting proteins (Dip) identified by tandem affinity purification or GFP-trap enrichment.

1	2	3	4	5	6	7	8
AN10456	DenA	Nedd8-specific protease	29.5	11	32.9	8	20.2
AN10946	DipA	putative ser/thr phosphatase	74.5	11	21.9	3	7.2
AN10352	*NOP56*	ribosome biogenesis	56.8	7	7.7	4	5.2
AN10614	*STM1*	ribosome binding	32.6	5	19.6	5	26
AN10557	*DED1*	putative RNA helicase	71.2	18	30	3	6
AN2068	*SCP160*	RNA binding and transport	141.5	5	4.6	5	5.1

The listed proteins were identified by LC-MS/MS analyses after enrichment of DenA-TAP by tandem affinity purification and by GFP-trap using fungal extracts with GFP fused to DenA. 1: Accession number, 2: Name/*S*. *cerevisiae* homolog, 3: Cellular function, 4: Molecular weight (kDa), 5: Number of unique peptides (TAP), 6: Sequence coverage in % (TAP), 7: Number of unique peptides (GFP), 8: Sequence coverage in % (GFP).

One of these putative DenA interaction proteins encoded by AN10946 was named DenA-interacting phosphatase A (DipA) since it is described as a putative serine/threonine phosphatase. However, DipA function has not been studied until now and its substrates for dephosphorylation are unknown. The co-purification of DipA with DenA suggests a possible role of the phosphatase in DenA dephosphorylation, stability control and fungal development. Other putative DenA interacting proteins are involved in translation such as proteins encoded by the homologs of yeast *NOP56* (AN10352) and *STM1* (AN10614). The NOP56 protein localizes in the nucleolus and processes rRNA for ribosome biogenesis [57]. The ribosome associated STM1 protein increases the quantity of ribosomes under nutritional stress conditions [58,59]. The fourth putative DenA interaction partner is encoded by AN10557 corresponding to yeast *DED1* and encodes an ATP dependent RNA helicase that is required for translation and shuttles between cytoplasm and nucleus [60]. This protein is related to a superfamily of DEAD-box proteins that contribute to cell cycle regulation and development [61,62]. The final putative DenA interacting protein encoded by AN2068 corresponds to yeast *SCP160* is a RNA transport and delivery protein which is associated with the nuclear envelope and the endoplasmatic reticulum. *SCP160* is involved in polarized growth and contains a potential nuclear export and nuclear localization sequence [63,64].

The co-purified DenA interacting proteins indicate a possible role of DenA in translation regulation and/or RNA transport processes. The interaction of DenA with the presumed phosphatase DipA was further examined to test whether it might be a link between destabilizing and stabilizing DenA phosphorylation sites during development.

### DipA including its metallophosphatase domain is conserved in the fungal kingdom

**Fig 5:**
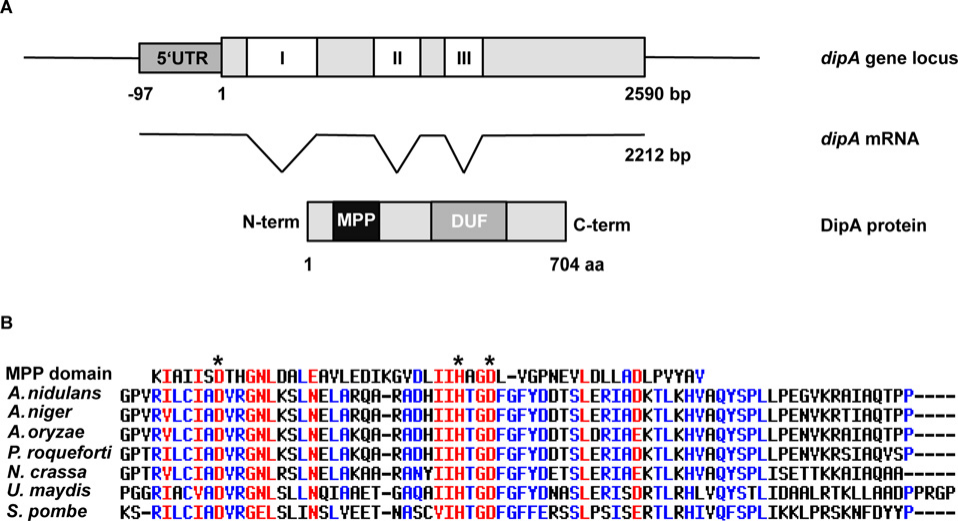
*dipA* gene locus and multiple alignment of its deduced metallophosphatase domain. **(A)** Schematic view of the *dipA* (AN10946) gene locus, transcript (mRNA) and deduced DipA protein. White boxes correspond to three introns (I, II, III). The metallophosphatase domain (MPP) is highlighted in black and the domain of unknown function (DUF) in grey. **(B)** Multiple alignment of MPP consensus sequence (cl13995) with DipA from *Aspergillus nidulans* and related proteins from other organisms including *Aspergillus niger*, *Aspergillus oryzae*, *Penicillium roqueforti*, *Neurospora crassa*, *Ustilago maydis* and *Schizosaccharomyces pombe*. Asterisks: putative active site D51, H73 and D76 residues. Red: high (90%), blue: low (50%) consensus values [56].

The *dipA* gene locus is located on chromosome IV and encodes a transcript of four exons interrupted by three introns with a length of 2590 bp (Fig 5A). The mRNA has a total length of 2212 bp including the coding sequence flanked by an upstream-untranslated region (5’UTR). The resulting DipA protein contains 704 aa and has a predicted mass of 74.5 kDa. BLAST search analyses [65] revealed that DipA is conserved within the fungal kingdom but is absent in higher eukaryotes such as plants or mammals. Highest amino acid sequence similarity was found in other *Aspergilli*. DipA related proteins of the industrial relevant strains *A*. *niger* and *A*. *oryzae* share 83% and 79% aa identity, respectively. Similar amino acid sequences were also present in *P*. *roqueforti* (75%), in the model organism *N*. *crassa* (59%), in the plant pathogen *Ustilago maydis* (51%) and the best match in *S*. *pombe* shares 51% aa identity to DipA. Two protein domains have been found when DipA sequence was analyzed using the conserved domain database of NCBI [66]. DipA contains a domain of unknown function (DUF2433; pfam10360), which ranges from aa 282 to 411. It is a conserved but uncharacterized region of a family of proteins found in fungi. The second domain belongs to metallophosphatases (MPPs), which represent a diverse superfamily of enzymes with an active site consisting of two metal ions, usually manganese, iron or zinc, that are coordinated by histidine (H), aspartate (D) and asparagine (N) residues. The MPP domain of DipA (cl13995) stretches from aa 45 to 96. Multiple alignments of MPP consensus sequence (pfam12850 [67]) with other organisms revealed that the MPP domain of DipA is conserved within the fungal kingdom (Fig 5B). Among the highest conserved residues are two aspartates (D51 and D76) and one histidine (H73) (marked with asterisks).

The identification of a MPP metallophosphatase domain in DipA implies that DipA is a catalytically active phosphatase and it is likely that the three conserved amino acids represent parts of the catalytic core.

### DipA phosphatase is required for septa positioning and asexual development

**Fig 6:**
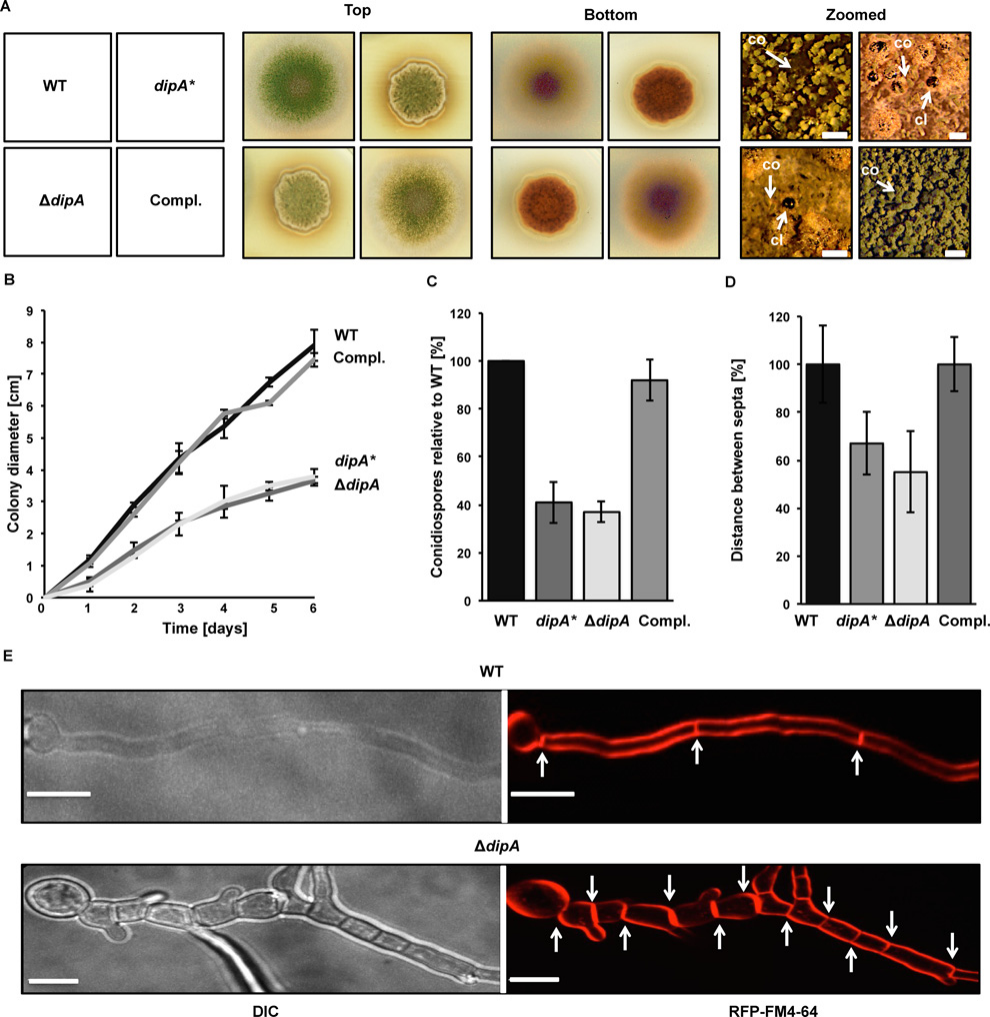
Phenotypical characterization of mutant strains lacking functional DipA. **(A)** Top and bottom view of point-inoculated wild type (WT), dipA* (codon exchange of catalytic core), *dipA* deletion (Δ*dipA*) and complementation (Compl.) strains incubated for three days under asexually development inducing conditions. Zoomed view represents binocular images of respective strains with asexual structures (conidiophores, co) and the sexual fruiting bodies (cleistothecia, cl) after seven days. Scale bar: 100 μm. **(B)** Colony diameter of point-inoculated asexually grown colonies measured for six days. The mean values with standard deviations derived from three independent experiments are shown. **(C)** Quantification of conidiospores after four days. The mean values with standard deviations from three independent experiments are shown. **(D)** Diagram illustrates distances between septa. Data derived from analyzing 70 hyphae of each strain. Shown are the mean values with standard deviations. **(E)** Fluorescence microscopy of hyphae of WT and *dipA* deletion strain. Membranes/septa were stained with FM4-64. White arrows are highlighting septa. Scale bar: 5 μm.

The three highly conserved amino acids aspartate at positions D51 and D76 as well as histidine H73 form the predicted catalytic core of the phosphatase DipA. The respective codons were mutated to determine whether an active DipA phosphatase domain is required for fungal development. The two aspartates D51 and D76 as well as the histidine H73 were substituted for alanine. The resulting dipA* strain carrying an inactive phosphatase was viable but revealed an aberrant growth and developmental phenotype when compared to wild type (Fig 6A). During asexual development the respective colony of the mutated DipA strain displayed a smaller size. In addition, the green pigmentation was reduced indicating decreased asexual development. A brownish stain surrounded the colony suggesting an impaired secondary metabolism.

A *dipA* deletion strain was generated to explore whether the loss of the entire protein displays similar phenotypes as the dipA* strain carrying an inactive phosphatase. Deletion of the *dipA* coding region resulted in a viable strain with the same defects in asexual development, colony morphology and secondary metabolism as observed for the dipA* mutant strain (Fig 6A). This implies that the inactive DipA phosphatase caused the various developmental phenotypes.

It is known that ilumination represses sexual development and promotes the asexual cycle of *A*. *nidulans* [68,69]. Detailed analyses of the asexual colonies of dipA* and *dipA* deletion strain after seven days in light revealed that they formed hardly any asexual conidiophores (co). Instead increased numbers of mature sexual fruiting bodies (cleistothecia, cl) were observed suggesting that lack of DipA results in a strain unresponsive to light (Fig 6A, zoomed). Reintegration of *dipA* at the endogenous locus in *dipA* deletion strain complemented the phenotype. Quantifications revealed that both strains lacking a functional DipA formed a colony that was reduced by half and produced only 40% of the conidiospores when compared to wild type or complementation strains (Fig 6B and 6C). This reduced conidiation in *dipA* deletion strain reflected fewer numbers of normal conidiophores. These data suggest that the activity of the phosphatase DipA is required for light signaling cascades which coordinate fungal development and secondary metabolism.

Hyphal morphology was studied to analyze whether impaired growth and conidia formation are caused by altered septa formation in the dipA* and *dipA* deletion strains. Fluorescence microscopy of vegetative hyphae with stained membranes, including septa, revealed that the strain with mutations in the putative catalytic core of DipA (dipA*) as well as a *dipA* deletion strain showed an increased septa positioning when compared to wild type and *dipA* complementation strain (Fig 6D and 6E). The distance between two septa was reduced to 67 ± 13% and 55 ± 17%, respectively, in strains with an altered *dipA* locus. Hyphal units, given as compartments flanked by one septum on either side, were observed with sizes reduced to 11 ± 4 μm. The width of the hypha was not altered in *dipA* deletion strain compared to wild type (d = 2.1 ± 0.2 μm). A reduction of the overall compartment volume in the mutant strain (41 ± 11 μm^3^) to approximately half of the wild type volume was observed (79 ± 18 μm^3^). These data indicate that DipA controls as well septa positioning as compartment volume.

Taken together, functional phosphatase DipA is required for light-inhibition of sexual development, asexual spore formation and accurate septa positioning to sustain fungal growth.

### DipA interacts and shuttles with the cytoplasmatic DenA subpopulation

**Fig 7:**
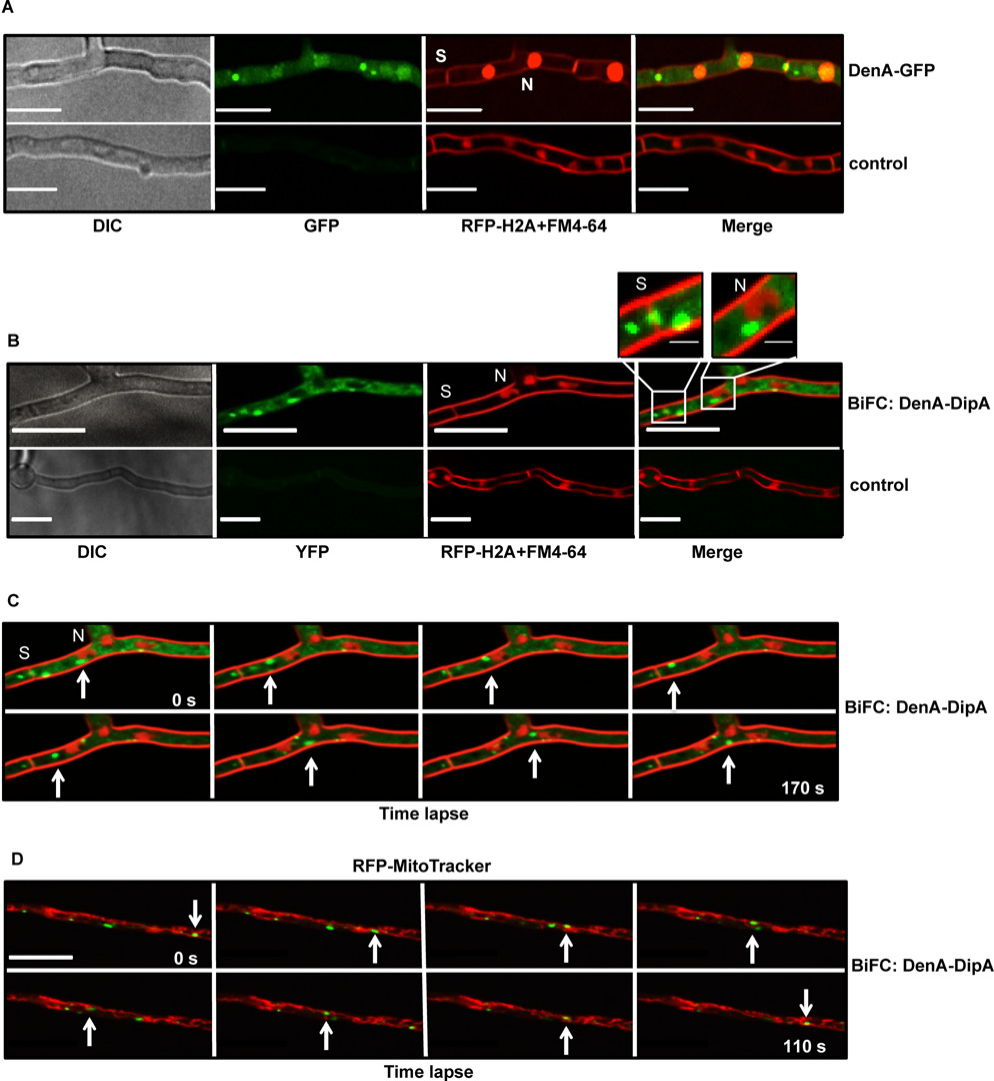
DenA-DipA cytoplasmatic movements *in vivo*. **(A)** Fluorescence microscopy of DenA-GFP subpopulations within vegetative hyphae located the protein in the nucleus (N), in the cytoplasm and there with a specific enrichment at septa (S). Control: wild type without GFP. **(B)** Bimolecular fluorescence studies (BiFC) of DenA (*denA*::*nyfp*) and DipA (*dipA*::*cyfp*) showed restricted interaction in the cytoplasm, at septa (S) and close to, but not inside nuclei (N). The septal and the nuclear regions are enlarged (white squares; scale bar: 1 μm). Control: strain co-expressing *denA*::*nyfp* and *cyfp*, respectively. **(C)** Dynamic co-transport of DenA-DipA between nuclei and septa in time lapse of bimolecular fluorescence strain *denA*::*nyfp*-*dipA*::*cyfp* over 170 seconds. White arrows mark a single interaction complex. **(D**) Time lapse microscopy over 110 seconds of *denA*::*nyfp*-*dipA*::*cyfp* with stained mitochondria (red) with a white arrow marking single DenA-DipA. Expressed *rfp*::*h2A* decorates nuclei, membranes were stained with FM4-64 and mitochondria with MitoTracker. Scale bar: 5 μm.

The protein half-life during development and the cellular distribution of the newly identified DenA interaction partner DipA were analyzed. Similar to DenA-GFP, DipA-GFP is a stable protein in vegetative hyphae and its protein levels decreased during advanced asexual development (S6A and S6B Fig). Fluorescence microscopy of DenA-GFP revealed that cellular DenA subpopulations are localized in the nucleus and in the cytoplasm and there predominantly at septa (Fig 7A). Analysis of the subcellular localization of DipA-GFP displayed a cytoplasmatic fraction that was transported through hyphae between nuclei and septa (S6C and S6D Fig). Bimolecular fluorescence complementation (BiFC) experiments with DenA-DipA supported the physical interaction as fluorescent binding complexes were observed in the cytoplasm near septa and in close proximity to, but not inside nuclei (Fig 7B). The overall distribution of DenA within the cell was unaffected by deletion of *dipA* (S7 Fig). Time lapse fluorescence microscopy experiments using the DenA-DipA BiFC strain revealed DenA-DipA dynamically moving through the cytoplasm. A bidirectional shuttling between nuclei and septa (Fig 7C, S1 Video and S2 Video) with interactions to the widespread structures of mitochondria (Fig 7D and S3 Video) was observed.

The BiFC study corroborates the physical interaction between DenA and DipA and reveals a dynamic shuttling of the DenA-DipA interaction complex which corresponds to the DipA-GFP localization in the cytoplasm and at septa that approaches nuclei and mitochondria.

### DipA controls the stability of cytoplasmatic DenA

**Fig 8:**
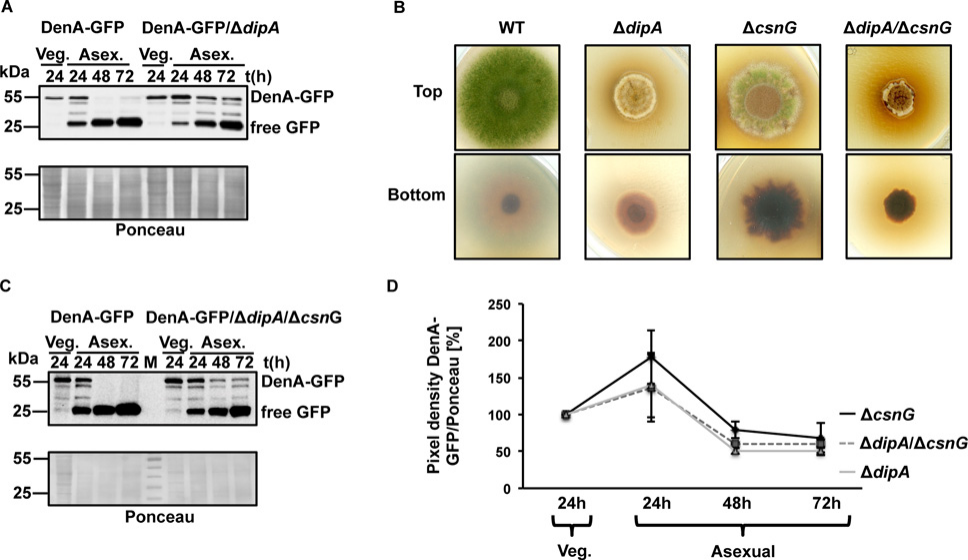
Comparative analyses of DenA protein levels in Δ*dipA*, Δ*csnG* and Δ*dipA*/Δ*csnG* double mutant strains. **(A)** DipA destabilizes DenA during illumination which induces asexual development in wild type. Western hybridization showed a stable DenA-GFP in wild type background at vegetative growth (Veg.) and an unstable protein during illumination induced asexual development (Asex). DenA-GFP in a Δ*dipA* strain is stable under the same conditions. **(B)** Top and bottom view of illuminated wild type colony (WT) developing asexual spores and *dipA*, *csnG* and combined *dipA*/*csnG* deletion strains under the same conditions. Equal amount of spores were point-inoculated and incubated for three days during illumination. **(C)** DenA-GFP protein stability in WT and *dipA/csnG* deletion background without the potential to produce asexual spores during illumination (Asex.). **(D)** Diagram of DenA-GFP protein levels in Δ*csnG*, Δ*dipA* and *dipA*/*csnG* double deletion strain from three independent experiments during vegetative growth (Veg.) and illumination (Asex.) for indicated time points. SDS gels were loaded with equal amounts of protein crude extract. Membranes were treated with GFP-antibody and stained with Ponceau as loading control and the GFP/Ponceau pixel ratio was calculated. The standard deviations are shown. DenA-GFP: 54.5 kDa and free GFP: 25 kDa.

The impact of the phosphatase DipA on DenA protein stability, which is controlled by phosphorylation, was analyzed. DenA-GFP was less unstable in a *dipA* deletion strain throughout the entire asexual development, whereas DenA-GFP in wild type carrying intact DipA was present at 24h of vegetative growth but was undetectable after 48h of asexual development (Fig 8A). These results indicate that DipA promotes degradation of DenA during asexual development inducing conditions. Since their interaction occurred in the cytoplasm it is likely that DipA destabilizes the cytoplasmatic DenA subpopulation.

A *dipA*/*csnG* double deletion strain lacking both proteins that destabilize DenA either in the nucleus (CsnG) or in the cytoplasm (DipA) was generated to analyze the combined impact on development and on DenA destabilization. The resulting strain exhibited a stronger phenotype than the one observed in the single deletions (Fig 8B). The growth defect as well as the accumulation of brownish compounds in the surrounding medium were increased in the double mutant when compared to either single deletion strains. The *dipA*/*csnG* double deletion strain could not further increase the amount of DenA in comparison to the single deletions of *dipA* and *csnG* after 72h of asexual development (Fig 8C and 8D).

In summary, DipA and CsnG might act independently on asexual development and reduce DenA amounts at different locations to the same extent suggesting a complex interrelation between the nuclear and cytoplasmatic DenA subpopulations.

## Discussion

**Fig 9:**
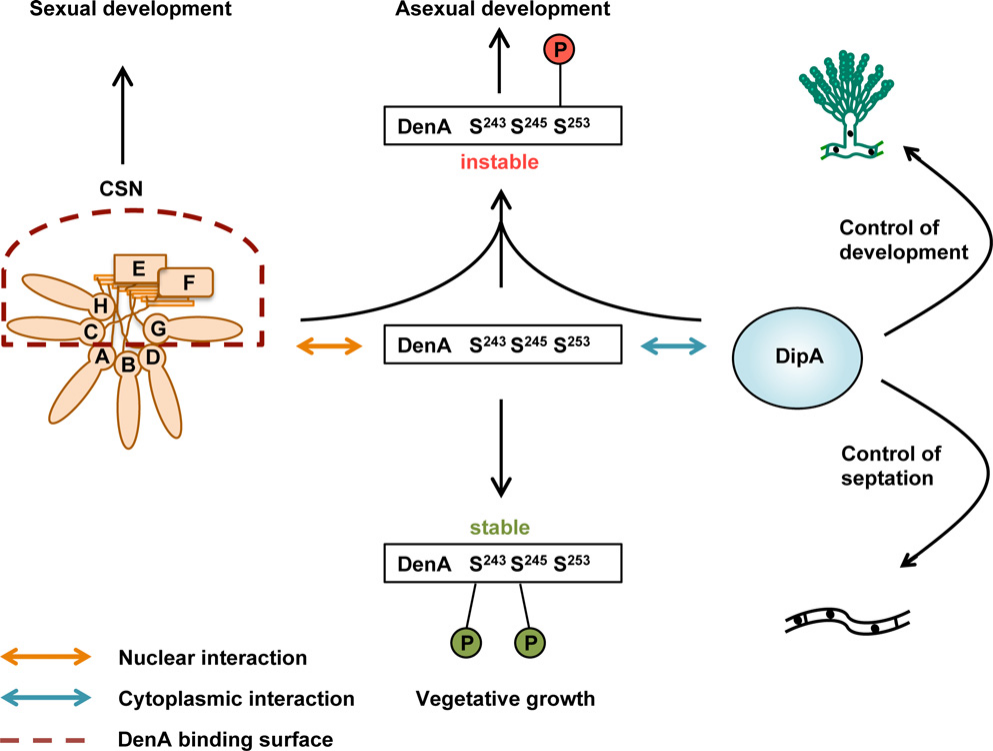
Dual DenA stability control during fungal development. The DenA deneddylase supports asexual development. DenA stability is regulated during fungal development by indicated dynamic phosphorylation and dephosphorylation events and the interaction with DipA (blue arrow) or CSN (orange arrow). Phosphorylation of DenA at serine residues S243 and S245 stabilizes the protein during vegetative growth. Stabilization during this growth phase and early stages of development is required for asexual spore formation induced by light. The cytoplasmatic phosphatase DipA controls the interval of septa positioning and is required for asexual development and light dependent inhibition of sexual differentiation. DenA interacts with DipA in the cytoplasm. This complex dynamically shuttles between nuclei and septa. The transition of a stable DenA to an unstable DenA variant coincides with the appearance of C-terminal S253 phosphorylation and dephosphorylation of S243 as well as S245 at later stages of development. Nuclear CSN is required for sexual development. DenA is destabilized by the five CSN subunits in the dashed frame which are localized in the nucleus and might form a common DenA binding surface.

The major finding of this study is that protein stability of the conserved DenA deneddylase is under a dual control located in the nucleus and in the cytoplasm, respectively. DenA can be as well stabilized as destabilized by different C-terminal phosphorylation tags which dynamically change during vegetative growth and fungal development. Nuclear DenA turnover control includes several but not all subunits of the COP9 signalosome whereas the cytoplasmatic turnover control in fungi depends on the novel and physically DenA interacting phosphatase DipA. The fungal DenA-DipA complex is highly mobile and can be found within the cytoplasm close to nuclei without entering and is enriched near septa. DipA is required for the fungal light response which normally promotes asexual development and reduces sexual development. The inhibition of sexual development is missing in strains without intact DipA. DipA is not only required for asexual spore formation but also for the correct spacing of septa within the fungal hyphae. DenA turnover depends on a complex interplay between two types of C-terminal serine phosphorylation sites which either stabilize or destabilize the protein. DenA has to be present and stabilized by phosphorylation during the initial phase of asexual development. Its stabilization is controlled by phosphorylation of at least two of the three C-terminal serine residues and is prerequisite to support asexual spore formation. A triple phosphorylated DenA protein is less unstable and still capable to support asexual development. Phosphorylation of only the most C-terminal destabilizing serine residue, which is presumably linked to cytoplasmatic DipA supported dephosphorylation of the two stabilizing serine sites, leads to DenA degradation (Fig 9).

Phosphorylation and protein destruction are often coupled processes [7,9]. Depending on the respective degron, addition of one or more phosphate groups to a protein can either stabilize the respective target (phospho-inhibited degron) or it becomes recognized by CRLs resulting in protein degradation (phosphodegron) [70,71]. DenA represents a stable protein during vegetative growth and presumably the establishment of developmental competence but converts into a destabilized variant later during development. DenA stability control correlates with fungal development and the phosphorylation of three serine residues located at its C-terminus at position S243, S245 and S253. The two phosphorylated serine residues at position S243 and S245, identified during vegetative growth, stabilize DenA. This implies that respective phosphorylation sites represent phospho-inhibited degrons which are important to prevent DenA degradation. A triple amino acid substitution of the identified serines to alanine, mimicking a constant dephosphorylated protein at these positions, caused this DenA variant to degrade earlier than wild type DenA during asexual development inducing conditions. This premature degradation of DenA coincided with impaired asexual development. The finding suggests that DenA has to be phosphorylated and stabilized at serines S243 and S245 to induce asexual development. After a defined period of vegetative growth, which ranges from 16h to 20h, hyphal cells reach a stage of developmental competence and become responsive to external stimuli [45,72–74]. DenA seems to support the transition from filamentous growth to asexual differentiation during the establishment and the evaluation process of developmental competence which results in the decision to induce asexual development. After this developmental transition DenA is absent and therefore presumably dispensable for further asexual development.

The importance to dephosphorylate DenA during fungal development is supported by the discovery of the DenA interacting phosphatase DipA. Deletion of the corresponding *dipA* gene resulted in an increased DenA stability during asexual development, suggesting that DipA uses DenA as substrate to remove the protective phosphates at S243 and S245. DipA as well as the three identified phosphorylation sites of DenA are conserved within the fungal kingdom and might represent an ancient mechanism to control the transition from filamentous growth to asexual differentiation.

Destabilization of DenA during later stages of asexual development depended on the presence of an additional serine at position S253 which also can be phosphorylated. A negative charge provided by an aspartate residue at this position produced an unstable DenA, whereas exchange of serine to alanine at this position resulted in a less unstable DenA. Negative charges at all three C-terminal DenA serine residues, provided by aspartate codon exchanges instead of the original serine residues, resulted in a less unstable protein compared to wild type. This observation suggests that S253 represents a phosphodegron and that DenA has to be dephosphorylated at positions S243 and S245 during asexual development before it can be destabilized by S253 phosphorylation at a later stage of differentiation.

DenA/DEN1 is a deneddylase that acts primarily on neddylated non-cullin proteins [16,38,39]. Stable DenA might be required to deneddylate so far unknown substrates as prerequisite to support the transition from vegetative growth to asexual development. The formation of reproductive structures such as asexual conidiospores requires high amounts of purines and pyrimidines representing essential components of RNA and DNA [75,76]. The impaired asexual spore formation of strains either lacking *denA* or displaying premature DenA degradation during limited pyrimidine supply hints to putative DenA substrates linked to pyrimidine metabolism.

The two deneddylases DenA/DEN1 and COP9 signalosome remove the ubiquitin-like protein Nedd8 from modified targets and are required for development in fungi, as well as in insects, plants and mammals [25,26,51,77]. In *A*. *nidulans* the two deneddylases are involved in different developmental pathways. DenA supports asexual development, which is promoted when the fungus reaches the soil surface and perceives light signals, whereas CSN is essential for sexual development [16,25]. Physical interaction between both deneddylases is conserved from fungi to human. Numerous pulldown experiments with differentially tagged DenA versions neither pulled out CSN subunits nor did CSN subunits recruit DenA [16]. This suggests a transient interaction between CSN and DenA. The nuclear CSN-DenA interaction might be part of a developmental control which delays asexual development and supports sexual differentiation and secondary metabolism e.g. in the soil in darkness. An *A*. *nidulans* strain without both of the deneddylases DenA and CSN grows predominantly vegetative and is highly impaired in multicellular development [16]. Lack of both deneddylases results in cullins with increased molecular weights which might represent polyneddylated cullins, suggesting a repressive function in the coordination of development for these cullin variants. Despite their different substrate specificity, both deneddylases possess catalytic activity towards cullins modified with multiple Nedd8 molecules. This indicates that both fungal deneddylases not only have distinct but also overlapping substrates. However, fungal and mammalian CSN seem to be different. The mammalian CSN has been reported to be unable to deneddylate multineddylated cullins [40], which might represent 1.5 Billion years of different evolution of the two groups.

*A*. *nidulans* strains deleted for *csn* encoding genes are unable to complete the sexual life cycle, whereas *denA* deletion strains are impaired in asexual differentiation [16,47]. Increased DenA isopeptidase activity was previously reported to compensate developmental defects caused by the absence of an isopeptidase responsible for deconjugating the ubiquitin-like protein SumO [78]. The present study revealed that increased amount of DenA counteracts the accumulation of neddylated proteins and impaired development caused by a defective COP9 signalosome, supporting their overlapping substrate specificity in fungi. The interference of both deneddylases suggests the necessity to degrade DenA under conditions where asexual development is negligible and sexual differentiation needs to be promoted by the CSN complex.

The presence of three PCI subunits (CsnC, CsnG, CsnH) and the two MPN subunits (CsnE, CsnF) destabilizes DenA, whereas the other three CSN subunits are irrelevant for DenA stability. The corresponding mammalian subunits CSN3, CSN5, CSN6, CSN7 and CSN8 provide a stability control function for the cyclin-dependent kinase inhibitor p27 [79,80]. The crystal structure of the human CSN complex revealed that it is composed of two organizational centers. The C-terminal α-helices of every subunit create a large bundle and the six subunits containing a PCI domain form a horseshoe-like structure [21]. According to the subunit composition, the five CSN subunits with destabilization effect on DenA are adjacent and probably form a common surface (Fig 9). A CSN holocomplex with activity towards neddylated cullins requires the presence of all subunits. Loss of a single CSN subunit results in an inactive CSN complex which is unable to deneddylate cullins [25,28]. A CsnC-E-F-G-H subcomplex is not yet present among several mini-CSNs which had been described in different systems [27,81–85]. The interaction between DenA and CSN was exclusively found inside the nucleus [16]. A transient nuclear mini-CSN might form a common interaction surface for DenA as prerequisite for nuclear DenA degradation and might even exhibit some nuclear deneddylase activity.

The localization data presented in this study imply that the interaction between DenA and CSN, which was previously found inside nuclei [16], is different from the cytoplasmatic DenA-DipA interaction complex. Different subpopulations of proteins present in separate compartments can display different degradation patterns [86,87]. The interaction with other proteins present in the different compartments might individually influence the stability of the respective protein [87,88]. It seems that cellular DenA protein levels in fungi are regulated by distinct mechanisms which mediate DenA stability control either by nuclear CSN or cytoplasmatic DipA (Fig 9). When both control mechanisms were impaired (e.g. by a *dipA/csnG* double deletion), the cellular DenA amount was similarly enriched compared to single deletion strains. This suggests that both stability checkpoints are communicating to each other in a yet unknown manner. Cytoplasmatic DenA is highly dynamic and shuttles between nuclei, mitochondria and septa. Transport of various cargoes, including mitochondria, vesicles, peroxisomes or vacuoles requires the cytoskeleton including tubulins and actins [89–91]. DenA interaction is not restricted to DipA but also includes possible transport proteins as the fungal counterpart of yeast SCP160 transporting RNA molecules within a cell. It is likely that association of DenA to DipA-dependent or even DipA-independent cellular transport processes might be part of the communication between different DenA stability control mechanisms in the nucleus and the cytoplasm, respectively.

The DenA interacting phosphatase DipA is required for septa positioning within fungal filaments which might be a prerequisite for asexual spore formation. Formation of septa is the equivalent of cytokinesis with the exception that septation leads to compartmentalization and mostly not complete cell separation [92]. Growing hyphae are normally partitioned into compartments separated by septa as cross walls that are formed at more or less regular intervals [93,94]. The formation of septa starts with the selection of the division site. The cytoskeleton plays an important role during septum formation where actin appears to be the first protein to mark the future septum site [95,96]. The second step in septa formation is the orderly assembly of >100 conserved proteins which are temporally and spatially coordinated to form a septum [97]. Septum formation finishes with dynamic constrictions of a contractile actomyosin ring, invagination of the plasma membrane and deposition of cell wall materials [94,96,98,99].

Fungal strains lacking either a functional DipA or the entire DipA encoding gene form increased amounts of septa. An active DipA phosphatase is therefore required to define the septation site and to ensure a correct septa interval. In mammals, yeast and filamentous fungi cell separation has been linked to posttranslational modifications, including phosphorylation. Septins, which represent a group of proteins that is involved in coordinating a variety of cellular processes at the site of septation, are controlled by phosphorylation [100,101]. The observed defects in septa formation of *dipA* mutant strains suggest an enrichment of phosphorylated substrates, such as septins, that cause the increased septation. Since DenA and DipA are co-transported in the cytoplasm and accumulate at septa it is still elusive whether these substrates are phosphorylated and/or neddylated and therefore are substrates of DipA and/or DenA, respectively. The analyzed *denA* mutant strains did not show obvious defects in septa formation in comparison to wild type, suggesting that DenA is presumably not directly involved in this phenotype. Cytoplasmatic DipA interacts at septa with phosphorylated DenA, which might point to the kinase which is the antagonist of DipA as another interesting player. Analyses of the DenA phosphorylation sites with a computational prediction tool [55] identified the fungal cyclin dependent cell cycle kinase Cdc2 as potential candidate responsible for phosphorylating DenA. The related human CDK2 interacts with enzymatically active CsnE/CSN5 [37], which could be the reason why this kinase was absent in our approach to identify DenA interacting proteins. *A*. *nidulans* NimX^Cdc2^ is the only mitotic cyclin-dependent protein kinase in the fungus and its controlled activity is required for septa formation [102,103]. The septation initiation network (SIN) represents a kinase cascade which coordinates mitosis and septum formation in filamentous fungi [93,104,105]. Increased NimX^Cdc2^ activity causes hyperseptated hyphae [102,106]. Since deletion of *dipA* in *A*. *nidulans* resulted in hyperseptation, it is possible that putative substrates of NimX^Cdc2^, which might belong to SIN, accumulate in their phosphorylated form in *dipA* deletion strain and cause the impaired septa formation.

Formation of septa is essential in filamentous ascomycetes for differentiation processes during developmental stages of the asexual and sexual cycle [107]. The asexual conidiophore forms conidiospores in a manner that is similar to diploid yeast pseudhohyphae formation and reminiscent of the process of septation. At the end of a central regulatory pathway asexual spores emerge on the conidiophore by continuous budding processes that give rise to long chains of conidiospores [69,73]. Reduced asexual spore formation as well as reduced growth of the *dipA* deletion strain observed in this study, might result from malfunctioning cell division. This is in accordance to phenotypical analyses of a plethora of phosphatase encoding genes in *A*. *nidulans*. Some of them are essential, others are required for normal growth or involved in mitosis, which supports the importance of phosphatase-mediated protein dephosphorylation in regulating cell cycle progression and development in fungi [108,109]. A *dipA* deletion strain is unresponsive to light and forms sexual structures in light combined with the accumulation of a brownish dye which is typical for impaired fungal secondary metabolism. Therefore, DipA seems to be required for fungal light response which normally promotes asexual development and reduces sexual development [73,110,111]. As development and secondary metabolism are often coordinated in fungi [45,112,113], DipA might represent an interesting target for the discovery of new secondary metabolites and respective gene clusters. The complex phenotype of *dipA* deletion strain suggests that developmental programs require the dynamic DipA-DenA interaction for the local dephosphorylation and/or deneddylation of multiple not yet identified substrates at various cytoplasmatic locations, including septa.

In summary, our study provides evidences for a dual mechanism controlling DenA stability in two cellular compartments which presumably reflects different DenA functions. It was unveiled that phosphorylation events trigger either stabilization or degradation of DenA. DipA might have different functions during vegetative growth and development. During vegetative growth DipA is required to protect DenA by dephosphorylating the destabilizing serine S253. Destabilizing phosphorylation of cytoplasmatic DenA might be delayed by the phosphatase DipA in this growth phase to overcome the initial phases of fungal differentiation during the window when the fungus establishes developmental competence and the future programs for differentiation are started. During later phases of development DipA might dephosphorylate the stabilizing serine residues S243 and S245, which results in subsequent phosphorylation of the destabilizing S253 that targets DenA for degradation. The orchestration of DenA phosphorylation might include nuclear kinases which are CSN associated kinases. The exact impact of NimX^Cdc2^ as a potential kinase for DenA remains to be studied. Future interest could focus on whether a similar phospho-regulation of DenA may account for higher eukaryotes similar to the interaction between DenA/DEN1 and CSN which is conserved from fungi to human [16,41].

DipA represents a fungal specific phosphatase which is absent in higher eukaryotes and is therefore an interesting candidate for a target to control fungal growth including pathogens which control host infection through the cell cycle [114–116]. The influence of DipA on cytokinesis may help to shed new light onto cell differentiation in plants and mammals and may extend the possibilities to manipulate pathways involved in cell cycle control. Deneddylases are major players in coordinating protein degradation and are linked to cancer development and neurodegenerative diseases [117–119]. The deneddylases CSN and DenA are connected by a tighter interrelation than previously expected which might provide interesting insights in understanding the dynamic cellular neddylation and deneddylation network and its consequences on development and disease.

## Materials and Methods

### Strains, media and growth conditions

*Escherichia coli* strain DH5α (Invitrogen) was cultivated in lysogeny broth (LB) medium (1% tryptophane, 0.5% yeast extract, 1% NaCl) [120] in the presence of 100 mg/ml ampicillin. Solid media contained 2% agar. *Aspergillus nidulans* strains used and generated in this study (Table 2) were grown in minimal medium (1% glucose, 2 mM MgSO_4_, 70 mM, NaNO_3_, 7 mM KCl, 11.2 mM KH_2_PO_4_, 0.1% trace element solution [121] pH5.5). Solid media contained 2% agar. Supplements such as 0.1% pyridoxine-HCl, 5 mM uridine, 5 mM uracil, 100 ng/ml pyrithiamine, 120 ng/ml nourseothricin and 10 μg/ml phleomycin were added when needed. For applying stress conditions 0.04 mM menadione or 0.015% SDS was added. Vegetative cultures were grown in liquid medium for 16-40h and asexual development was induced either by distributing equal amounts of spores on solid medium or in case of developmental synchronization by transferring pregrown vegetative mycelia onto solid medium. Plates were incubated at 30°C or 37°C during constant white light exposure. Samples for protein extraction were harvested at the indicated time points.

**Table 2:** *A*. *nidulans* strains constructed and used in this study.

Strain	Genotype	Reference
TNO2a3	*pyrG89*;*pyroA4*;*argB2*;*ΔnkuA*::*argB*	[122]
AGB152	*pyrG89*;*pyroA4*	[47]
AGB160	*pyrG89*;*pyroA4;pyr4*^+^	[47]
AGB195	*pyrG89*;*pyroA4*;Δ*csnD*::*pyr4*^*+*^	[47]
AGB209	*pyrG89*;*pyroA4*;Δ*csnE*::*pyr4*^*+*^	[47]
AGB223	*pyrG89*;*pyroA4*;Δ*csnA*::*pyr4*^*+*^	[29]
AGB238	*pyrG89*;*pyroA4*;*ΔcsnB*::*pyrG*^*+*^_*af*_	[29]
AGB316	*pyrG89*;*pyroA4*;*ΔdenA*::*pyr4*^+^	[16]
AGB318	*pyrG89*;*pyroA4*;*ΔdenA*::*pyr4*^+^;*denA*^+^;*phleo*^R^	[16]
AGB466	*pyrG89*;*pyroA4*;*ΔnkuA*::*argB*;*ΔcsnE*::*ptrA*^R^	[16]
AGB551	*pyrG89*;*pyroA4*	[123]
AGB596	^P^*gpdA*::*gfp*::*phleo*^R^;*pabaA1*	[123]
AGB630	*pyrG89*;*pyroA4*;*ΔdenA*::*pyr4*^+^;	[16]
	^P^*niaD*::*cyfp*::*niaD*^T^/^P^*niiA*::*denA*^cDNA^::*nyfp*::*niiA*^T^;*ptrA*^R^	
AGB631	*pyrG89*;*pyroA4*;Δ*csnG*::*ptrA*^R^	[25]
AGB632	*pyrG89*;*pyroA4*;Δ*csnE*::*ptrA*^R^;*ΔdenA*::*pyr4*^+^	[16]
AGB634	*pyrG89*;*pyroA4*;^P^*denA*::*denA*::*gfp*::*nat*^R^:: *denA*^T^	[16]
AGB635	*pyrG89*;*pyroA4*;Δ*csnA*::*pyr4*^*+*^;	This study
	^P^*denA*::*denA*::*gfp*::*nat*^R^::*denA*^T^	
AGB636	*pyrG89*;*pyroA4*;^P^*denA*::*denA*::*ctap*::*nat*^R^::*denA*^T^	This study
AGB640	*pyrG89*;*pyroA4*;^P^*denA*::*denA*::*gfp*::*nat*^R^::*denA*^T^;	[16]
	^P^*gpdA*::*mrfp*::*h2A*::*hisB*^T^;*pyrG*^*+*^_*af*_	
AGB641	*pyrG89*;*pyroA4*;*mrfp*::*h2A*;*phleo*^R^	[16]
AGB649	*pyrG89*;*pyroA4*;Δ*csnE*::*pyr4*^*+*^;	This study
	^P^*denA*::*denA*::*gfp*::*nat*^R^::*denA*^T^	
AGB667	*pyrG89*;*pyroA4*;*sasA*::*ctap*::^P^*gpdA*::*nat*^R^	[124]
AGB708	*pyrG89*;*pyroA4*;^P^*denA*::*denA*::*gfp*::*nat*^R^::*denA*^T^;	[16]
	*ΔcsnG*::*ptrA*^R^;^P^*gpdA*::*mrfp*::*h2A*::*hisB*^T^;*pyrG*^*+*^_*af*_	
AGB718	*pyrG89*;*pyroA4*;*ΔcsnC*::*pyrG*^*+*^_*af*_	[25]
AGB720	*pyrG89*;*pyroA4*;*ΔcsnF*::*pyrG*^*+*^_*af*_	[25]
AGB722	*pyrG89*;*pyroA4*;*ΔcsnH*::*pyrG*^*+*^_*af*_	[25]
AGB959	*pyrG89*;*pyroA4*;*ΔdenA*::*pyr4*^+^;	This study
	^P^*niaD*::*dipA*^cDNA^::*cyfp*::*niaD*^T^/	
	^P^*niiA*::*denA*^cDNA^::*nyfp*::*niiA*^T^;*ptrA*^R^	
AGB960	*pyrG89*;*pyroA4*;*ΔdipA*::*ptrA*^R^	This study
AGB961	*pyrG89*;*pyroA4*;*ΔdenA*::*pyr4*^+^;	This study
	^P^*niaD*::*dipA*^cDNA^::*cyfp*::*niaD*^T^/	
	^P^*niiA*::*denA*^cDNA^::*nyfp*::*niiA*^T^;	
	*ptrA*^R^;^P^*gpdA*::*mrfp*::*h2A*::*hisB*^T^;*phleo*^R^	
AGB962	*pyrG89*;*pyroA4*; ^P^*niiA*::*denA*::*niiA*^T^;*pyrG*^*+*^_*af*_	This study
AGB963	*pyrG89*;*pyroA4*;^P^*niiA*::*denA*::*niiA*^T^;*pyrG*^*+*^_*af*_;	This study
	*ΔcsnE*::*ptrA*^R^	
AGB964	*pyrG89*;*pyroA4*;^P^*niiA*::*denA*::*niiA*^T^;*pyrG*^*+*^_*af*_;	This study
	*ΔcsnG*::*ptrA*^R^	
AGB965	*pyrG89*;*pyroA4*;*ΔdipA*::*ptrA*^R^;	This study
	^P^*denA*::*denA*::*gfp*::*nat*^R^::*denA*^T^	
AGB966	*pyrG89*;*pyroA4*;	This study
	^P^*denA*::*denA*^S243A-S245A-S253A^::*gfp*::*nat*^R^::*denA*^T^	
AGB967	*pyrG89*;*pyroA4*;	This study
	^P^*denA*::*denA*^S243D-S245D-S253D^::*gfp*::*nat*^R^::*denA*^T^	
AGB968	*pyrG89*;*pyroA4*;^p^*dipA*::*dipA*::*gfp*::*nat*^R^::*dipA*^T^	This study
AGB969	*pyrG89*;*pyroA4*;*ΔcsnB*::*pyrG*^*+*^_*af*_;	This study
	^P^*denA*::*denA*::*gfp*::*nat*^R^::*denA*^T^	
AGB970	*pyrG89*;*pyroA4*;*ΔcsnC*::*pyrG*^*+*^_*af*_;	This study
	^P^*denA*::*denA*::*gfp*::*nat*^R^::*denA*^T^	
AGB971	*pyrG89*;*pyroA4*;Δ*csnD*::*pyr4*^*+*^; ^P^*denA*::*denA*::*gfp*::*nat*^R^::*denA*^T^	This study
AGB972	*pyrG89*;*pyroA4*;*ΔcsnF*::*pyrG*^*+*^_*af*_;	This study
	^P^*denA*::*denA*::*gfp*::*nat*^R^::*denA*^T^	
AGB973	*pyrG89*;*pyroA4*;*ΔcsnH*::*pyrG*^*+*^_*af*_; ^P^*denA*::*denA*::*gfp*::*nat*^R^::*denA*^T^	This study
AGB974	*pyrG89*;*pyroA4*;*ΔdipA*::*ptrA*^R^; ^P^*denA*::*denA*::*gfp*::*nat*^R^::*denA*^T^;*ΔcsnG*::*pyroA*_*af*_	This study
AGB975	*pyrG89*;*pyroA4*;^P^*denA*::*denA*^S253A^::*gfp*::*nat*^R^::*denA*^T^	This study
AGB976	*pyrG89*;*pyroA4*;^P^*denA*::*denA*^S253D^::*gfp*::*nat*^R^::*denA*^T^	This study
AGB977	*pyrG89*;*pyroA4*;^P^*denA*::*denA*::*gfp*::*nat*^R^::*denA*^T^;	This study
	^P^*dipA*::*dipA*^D51A;H73A;D76A^::6x*his*::*pyrG*::*dipA*^T^	
AGB978	*pyrG89*;*pyroA4*;*ΔdenA*::*pyr4*^+^;	This study
	^P^*niaD*::*dipA*^cDNA^::*cyfp*::*niaD*^T^/	
	^P^*niiA*::*denA*^cDNA S243A-S245A-S253A^::*nyfp*::*niiA*^T^;*ptrA*^R^	
AGB979	*pyrG89*;*pyroA4*;*ΔdenA*::*pyr4*^+^; ^P^*niaD*::*dipA*^cDNA^::*cyfp*::*niaD*^T^/	This study
	^P^*niiA*::*denA*^cDNA S243D-S245D-S253D^::*nyfp*::*niiA*^T^;*ptrA*^R^	
AGB980	*pyrG89*;*pyroA4*;^p^*dipA*::*dipA*::*gfp*::*nat*^R^::*dipA*^T^; ^P^*gpdA*::*mrfp*::*h2A*::*hisB*^T^;*phleo*^R^	This study
AGB981	*pyrG89;pyroA4*;*ΔdipA*::*ptrA*^R^;	This study
	^P^*denA*::*denA*::*gfp*::*nat*^R^::*denA*^T^;	
	^P^*gpdA*::*mrfp*::*h2A*::*hisB*^T^;*phleo*^R^	

P = promotor; T = terminator; R = resistance; *nat* = nourseothricine; *ptrA* = pyrithiamine; *phleo* = phleomycine; af = *Aspergillus fumigatus*

### Construction of *denA* overexpression strains

The *csnG* deletion cassette was generated by PCR mediated fusion [125] of the *csnG* flanking regions to the *ptrA* resistance cassette [126]. 1.2 kb 5’ flanking region of *csnG*, containing a downstream overhang for the *ptrA* cassette was amplified with primers MC125/MC126. PCR with primers MC129/MC130 generated a 2.1 kb fragment of the *csnG* 3’ flanking region with an upstream overhang for *ptrA*. The *ptrA* cassette with overhangs for each *csnG* flanking region was amplified from pSK409 with primers MC127/MC128. All three PCR fragments were assembled in a fusion PCR reaction with primers MC125/MC130. The deduced fragment was introduced into pJET1.2 resulting in plasmid pME3887. *csnG* deletion strain was obtained by transformation of a 5.7 kb *Xho*I fragment, excised from pME3887 into AGB152 resulting in AGB631. For overexpression of *denA* in different genetic backgrounds we transformed plasmid pME4068 ectopically into AGB152, AGB631 and AGB466 resulting in AGB962, AGB964 and AGB963. Ectopical integration was verified by Southern analysis confirming similar numbers of ectopically integrated copies in each strain. qRT-PCR using primers 5‘RT MC1/3’RT MC2 and 5‘RT H2A/3’RT H2A was performed to analyze similar *denA* expression levels.

### Strain construction of DenA-GFP in *csn* deletion strains

*csn* deletion strains carrying a C-terminal fusion of DenA with GFP under control of the native *denA* promotor were obtained by transforming a 5.5 kb *denA-gfp-nat*^R^ cassette, digested with *Cla*I/*Not*I from pME3900, into *csn* deletion strains AGB223, AGB238, AGB718, AGB195, AGB209, AGB720, and AGB722. The resulting strains were named AGB635, AGB969, AGB970, AGB971, AGB649, AGB972 and AGB973. Southern hybridization proved homologous recombination of the *denA-gfp-nat*^R^ construct at the endogenous locus.

### Construction of C-terminal *tap* tag fusion of *denA*

Primers ÖZG209/ÖZG192 were used to amplify a combined fragment of *ctap* and the *nat* resistance cassette (*ctap*::*nat*^*R*^) from strain AGB667. The 5’ flanking region and the *denA* ORF with 3’ sequence overhang to the *ctap* sequence was obtained with primers MC177/MC178 using genomic DNA as template. The 3’ flanking region of *denA* with 5’ sequence overhang for the *nat*^R^ resistance cassette was amplified with primers MC175/MC176. All fragments were fused together with primers MC1/MC2 by PCR mediated fusion [125] and subsequently ligated into pJET1.2/blunt resulting in plasmid pME3901. The strain used for identification of DenA interacting proteins and of DenA phosphorylation-sites by tandem affinity purification was achieved by excising plasmid pME3901 with *Xho*I and transforming the 4.5 kb *denA*::*ctap*::*nat*^*R*^ fusion cassette into AGB152 resulting in AGB636. Southern hybridization verified integration at the endogenous locus.

### Construction of strains with amino acid substituted DenA phosphorylation-sites

For strains containing a DenA-GFP version with substituted amino acids mimicking either a constant phosphorylated or a constant dephosphorylated status we exchanged the respective serine residues to alanine or aspartate, respectively. Single amino acid substituted DenA-GFP versions with an exchanged serine S253 to alanine or aspartate were obtained by using primers MC1/JS201 to amplify a 2.2 kb fragment containing the 5`flanking region as well as the first 1116 bp of *denA* ORF. With primers JS202/MC2 or JS203/MC2 serine S253 was substituted either to alanine or to aspartate, respectively. The amplicons contained a C-terminally located GFP fused to *nat* resistance under control of the constitutive *gpdA* promotor and followed by 3’ flanking region. Single fragments were fused by PCR with primers MC1/MC2 and the resulting 5.5 kb cassette was subcloned into pJET1.2/blunt vector giving plasmids pME4406 and pME4407, respectively. After digestion with *Cla*I/*Not*I the constructs were integrated at the native *denA* locus of TNO2a3. Southern hybridization of the generated strains AGB975 and AGB976 confirmed the integrity of the constructed strains.

Construction of DenA-GFP strains with three substituted DenA phosphorylation-sites were obtained in a similar way. Primers MC1/JS148 were used to amplify a 2.2 kb fragment containing the 5`flanking region as well as the first 1086 bp of *denA* ORF. A fragment containing S243, S245 and S253 substituted to alanine was obtained by PCR with primers JS150/JS162. Similarly, primers JS158/JS163 were used to substitute S243, S245 and S253 to aspartate. The C-terminal part of *denA* in frame with downstream located GFP fused to *nat* resistance under control of the constitutive *gpdA* promotor and followed by 3’ flanking region was amplified with primers JS167/MC2. All fragments were fused by PCR [125] using primers MC1/MC2 and ligated into pJET1.2/blunt vector resulting in pME4404 and pME4405, respectively. The 5.5 kb cassette was excised with *Cla*I/*Not*I and transformed into TNO2a3 giving strains AGB966 and AGB967. Southern hybridization verified integration at the endogenous locus.

Plasmids with mutations in the codons for the DenA phosphorylation-sites were constructed to analyze the consequences of amino acid substituted phosphorylation-sites on fungal phenotype. These plasmids were ectopically integrated into the *denA* deletion strain. cDNA of *dipA* connected with a linker to the C-terminal part of *yfp* (*cyfp*) was amplified from pME4402 with primers JS91/JS96. The 2.4 kb fragment was ligated into the *Swa*I restriction site of pSK409, resulting in plasmid pME4408. PCR with the primers MC30/JS204 were used to amplify cDNA of *denA* to substitute S243, S245 and S253 to alanine. The N-terminal half of *yfp* (*nyfp*) was amplified with primers JS205/MC171. Both fragments were fused with primers MC30/MC171 and the product with a size of 1.3 kb was ligated into the *Pme*I restriction site of pME4408, giving plasmid pME4409. Primers MC30/JS206 were used to substitute S243, S245 and S253 to aspartate. The obtained 776 bp fragment was fused to the N-terminal part of *yfp* (*nyfp*). The resulting product with a size of 1.3 kb was ligated into the *Pme*I restriction site of pME4408, giving plasmid pME4410. Transformation of pME4409 and pME4410 in *denA* deletion strain AGB316 resulted in strains AGB978 and AGB979, respectively. Ectopical integration of pME4409 and pME4410 was verified with Southern analyses using *Bg*lI.

### BiFC plasmid and strain construction for DenA-DipA interaction studies

Bimolecular fluorescence (BiFC) studies were performed with each half of a split *yfp* fused to proteins to test for interaction. Fusion proteins were under control of a bidirectional nitrate promotor. cDNA of *denA* connected with a linker to the N-terminal part of *yfp* (*nyfp*) was amplified from pME3897 with primers MC171/MC30. The 1.2 kb fragment was ligated into the *Pme*I restriction site of pSK409, giving plasmid pME4401. The C-terminal part of *yfp* (*cyfp*) and the appropriate linker was obtained using pME3897 as template and primers JS90/JS91 and JS89/JS92, respectively. Both fragments were fused with primers JS89/JS91 and the product with a size of 312 bp was ligated into the *Swa*I restriction site of pME4401 resulting in plasmid pME4418. *dipA* cDNA with a size of 2.1 kb was amplified with primers JS96/JS97 from TNO2a3 cDNA pool. This fragment was fused with the *cyfp*-linker fragment, obtained from plasmid pME4418 by PCR with primers JS93/JS91, using PCR mediated fusion with primers JS96/JS91. The resulting 2.4 kb product was ligated into the *Swa*I restriction site of pME4401, giving plasmid pME4402. AGB316, carrying a *denA* deletion construct with a pyrimidine marker causing a synthetic phenotype, was used to transform pME4402. Transformation of pME4402 into AGB316 resulted in strain AGB959. The phenotype was complemented suggesting a functional DenA protein. Ectopical integration was examined with Southern analyses. For visualizing nuclei, the plasmid pME3857 was transformed into AGB959 and ectopic integration of the *mrfp*::*h2A* construct was verified by microscopy. The resulting strain was named AGB961.

### Plasmid and strain construction of dipA* strain

The three amino acids D51, H73 and D76 which belong to the predicted catalytic core of DipA were substituted to alanine. PCR with primers JS219/JS221 were used to amplify the 5’ flanking region of AN10946 and to mutate D51. To assure that the in close proximity lying neighboring gene AN10959 is not disturbed, the amplified 3.1 kb fragment included the gene locus of AN10959. Primers JS222/JS223 were used to obtain a 184 bp fragment with substituted H73 and D76. PCR with primers JS224/JS220 amplified a fragment of 5 kb containing the C-terminal *dipA* fused to GFP and a *nat*-marker under control of the constitutive *gpdA* promotor followed by the 3’ flanking region of AN10946. PCR mediated fusion of all three fragments using primers JS219/JS220 resulted in an 8.2 kb product, that was ligated into pJET1.2/blunt vector giving plasmid pME4419. pME4419 was used as a template for primers JS219/JS228 to amplify a fragment of 5.3 kb containing the mutated *dipA* gene fused to 6xHis-Tag. PCR with JS225/JS226 resulted in amplified *pyrG*-marker with a size of 2 kb. The 3’ flanking region had a size of 900 bp and was generated with primers JS227/JS220. Fusion PCR with all three fragments using primers JS219/JS220 resulted in an 8.2 kb product which was ligated into pJET1.2/blunt vector giving plasmid pME4420. A 7.4 kb cassette was excised with *Psi*I/*Sna*BI and transformed into AGB634 resulting in AGB977.

### Plasmid and strain construction of the *dipA* deletion and complementation strain

For deleting the *dipA* gene locus (AN10946) the 5’ flanking region was amplified with primers JS131/JS132. To assure that the in close proximity lying neighboring gene AN10959 is not disturbed, the amplified 2.6 kb fragment included the gene locus of AN10959. The 3’ flanking region had a size of 1 kb and was obtained by PCR with primers JS135/JS136. Both products had an overhang to the *ptrA* marker [126] which was amplified with primers JS133/JS134. The *dipA* deletion cassette was fused by PCR [125] using primers JS131/JS136 and subcloned into pJET1.2/blunt vector resulting in plasmid pME4399. The 5.6 kb cassette was excised with *Eco*RV and transformed into AGB551 giving strain AGB960. Southern hybridization confirmed deletion of AN10946.

For complementation the 5’ upstream fragment including the ORF of *dipA* with a size of 5.3 kb was amplified with primers JS171/JS168. It contained a linker and an overhang for GFP cassette. The 2.1 kb *gfp* cassette fused to *nat* resistance under control of the constitutive *gpdA* promotor was obtained by PCR with primers ÖZG207/JS169. The 3’ flanking region with an overhang to the *nat* resistance was amplified with primers JS170/JS172. By using primers JS171/JS172 the three fragments were fused and subcloned into pJET1.2/blunt vector giving plasmid pME4400. The 8.2 kb complementation cassette was excised with *Nde*I and transformed into *dipA* deletion strain AGB960 resulting in strain AGB968. The integrity of the complementation cassette was confirmed by Southern hybridization. For visualizing nuclei, the plasmid pME3857 was transformed into AGB968 and ectopic integration of the *mrfp*::*h2A* construct was verified by microscopy. The resulting strain was named AGB980.

### Strain construction of DenA-GFP in *dipA* deletion and *dipA*/*csnG* double deletion strain

*dipA* deletion strain AGB960 was used to transform a 5.5 kb *denA-gfp-nat*^R^ cassette excised with *Cla*I/*Not*I from pME3857 resulting in strain AGB965. Southern hybridization and microscopy proved integration of the construct at the native *denA* locus. For visualizing nuclei, the plasmid pME3857 was transformed into AGB965 and ectopic integration of the *mrfp*::*h2A* construct was verified by microscopy. The resulting strain was named AGB981.

For deleting *csnG*, the 2.2 kb 5’flanking region was amplified with primers JS180/JS181. The 1.9 kb 3’ flanking region was amplified with primers JS184/JS185. The 2 kb *pyroA* fragment was obtained from template pME3979 by the use of primers JS182/JS183. All three fragments were fused together with primers JS180/JS185 resulting in a 6 kb *csnG* deletion cassette which was ligated into pJET1.2/blunt vector, giving plasmid pME4403. The construct was excised with *Bgl*II and transformed into AGB965 resulting in strain AGB974. Deletion of *csnG* as well as integrity of the *denA-gfp-nat*^R^ cassette was confirmed by Southern hybridization.

### Molecular methods

Transformation of *E*. *coli* and *A*. *nidulans* was described earlier [127,128]. Plasmids used for transformation are listed in Table 3. Primers used in this study are listed in Table 4. Isolation of plasmid DNA from *E*. *coli* was carried out using the Qiagen Plasmid MINI-Kit according to user’s manual. Genomic DNA from *A*. *nidulans* was isolated as described previously [129]. Southern hybridization was performed using the non-radioactive AlkPhos Direct Labeling and Detection System referring to manufacturer’s instructions (GE Healthcare).

**Table 3:** Plasmids constructed and used in this study.

Plasmid	Description	Reference
pJET1.2/blunt	cloning vector	Thermo Scientific GmbH
pSK409	^P^*niaD*::*niaD*^T^/ ^P^*niiA*::*niiA*^T^;*ptrA*^R^	Kindly provided by S. Krappmann
pME3160	^P^*niaD*::*niaD*^T^/ ^P^*niiA*::*niiA*^T^;*pyrG*^*+*^_*af*_	[130]
pME3857	^P^*gpdA*::*mrfp*::*h2A*::*hisB*^T^*;phleo*^R^	[123]
	in pBlueII SK+	
pME3887	5’^csnG^::*ptrA*^R^::3’^csnG^ in pJET1.2/blunt	This study
pME3897	^P^*niaD*::*csnG*^cDNA^::*cyfp*::*niaD*^T^/	Kindly provided by M. Christmann
	^P^*niiA*::*denA*^cDNA^::*nyfp*::*niiA*^T^; *pyrG*^*+*^_*af*_	
pME3900	5’^*denA*^::*denA*::*gfp*::*nat*^R^::3’^*denA*^	[16]
	in pJET1.2/blunt	
pME3901	5’^*denA*^::*denA*::*ctap*::*nat*^R^::3’^*denA*^	This study
	in pJET1.2/blunt	
pME3979	5’^*pyroA*^::*pyroA*_*af*_::3’^pyroA^ in pJET1.2/blunt	Kindly provided by R. Harting
pME4068	gDNA *denA* in pME3160 (*Swa*I)	[78]
pME4399	AN10959::5’^*dipA*^::*ptrA*^R^::3’^*dipA*^	This study
	in pJET1.2/blunt	
pME4400	AN10959::5’^*dipA*^::*dipA*::*gfp*::*nat*^R^::3’^*dipA*^	This study
	in pJET1.2/blunt	
pME4401	^P^*niaD*::*niaD*^T^/ ^P^*niiA*::*denA*^cDNA^::*nyfp*::*niiA*^T^;*ptrA*^R^	This study
pME4402	^P^*niaD*::*dipA*^cDNA^::*cyfp*::*niaD*^T^/	This study
	^P^*niiA*::*denA*^cDNA^::*nyfp*::*niiA*^T^;*ptrA*^R^	
pME4403	5’^*csnG*^::*pyroA*_*af*_::3’^*csnG*^ in pJET1.2/blunt	This study
pME4404	5’^*denA*^::*denA*^S243A-S245A-S253A^::*gfp*::*nat*^R^::3’^*denA*^ in pJET1.2/blunt	This study
pME4405	5’^*denA*^::*denA*^S243D-S245D-S253D^::*gfp*::*nat*^R^::3’^*denA*^ in pJET1.2/blunt	This study
pME4406	5’^*denA*^::*denA*^S253A^::*gfp*::*nat*^R^::3’^*denA*^	This study
	in pJET1.2/blunt	
pME4407	5’^*denA*^::*denA*^S253D^::*gfp*::*nat*^R^::3’^*denA*^	This study
	in pJET1.2/blunt	
pME4408	^P^*niaD*::*dipA*^cDNA^::*cyfp*::*niaD*^T^/	This study
	^P^*niiA*:: *niiA*^T^;*ptrA*^R^	
pME4409	^P^*niaD*::*dipA*^cDNA^::*cyfp*::*niaD*^T^/	This study
	^P^*niiA*::*denA*^cDNA S243A-S245A-S253A^::*nyfp*::*niiA*^T^;	
	*ptrA*^R^	
pME4410	^P^*niaD*::*dipA*^cDNA^::*cyfp*::*niaD*^T^/	This study
	^P^*niiA*::*denA*^cDNA S243D-S245D-S253D^::*nyfp*::*niiA*^T^;	
	*ptrA*^R^	
pME4418	^P^*niaD*::*cyfp*::*niaD*^T^/ ^P^*niiA*::*denA*^cDNA^::*nyfp*::*niiA*^T^;*ptrA*^R^	This study
pME4419	*AN10959*::5’^*dipA*^::*dipA*^D51A-H73A-D76A^::	This study
	*gfp*::*nat*^R^::3’^*dipA*^ in pJET1.2/blunt	
pME4420	*AN10959*::5’^*dipA*^::*dipA*^D51A-H73A-D76A^::	This study
	6x*his*::*pyrG*::3’^*dipA*^ in pJET1.2/blunt	

P = promotor; T = terminator; R = resistance; *nat* = nourseothricine; *ptrA* = pyrithiamine; *phleo* = phleomycine; af = *Aspergillus fumigatus*

**Table 4:** Oligonucleotides used in this study.

Designation	5’ – sequence – 3'
MC1	GTAATCGATGTCATCGCTGAAAAGGG
MC2	CCTGCGGCCGCTCTACATGGGTATGACTAGAG
MC30	CAATGCGCGACGGAGGGCTAGG
MC125	TACCGAGACTATCAAGGGAC
MC126	CATCTAGGCCTCGTGGCTGGTGTTGTTGG
MC127	ACCAGCCACGAGGCCTAGATGGCCTCTTGC
MC128	ACAATGAGATGGGCCACTCAGGCCAATTGA
MC129	CTGAGTGGCCCATCTCATTGTACGGTTCAGG
MC130	TACTCGAGCGCTGCAAAACGAAACACCA
MC171	TCACATGATATAGACGTTGTGGCT
MC175	ATCAAGACCCGAGGCAATTTGAC
MC176	CAGGCGCTCTACATGAGCATGCCCTGCCCCTGATAGTTGGCCCGACCGCTTCTAC
MC177	CTTTTTCCATCTTCTCTTACCACCGCTACCACCCTCAATACGCGGCGGACTCCTC
MC178	ATCGCCGAATCAGAGGCCAATGT
5’RT MC1	GACGATTCACCAACCCAAGAGA
3’RT MC2	CTACCTTCCAGCCACCCAAACT
5’RT H2A	TGCGGTCGTGTTAAGCGTTT
3’RT H2A	CGGATGGCAAGCTGTAGGTG
JS89	ATGCGCCCGGCCTGCAAG
JS90	AACAGAAGGTCATGAACCACGCCGACAAGCAGAAGAACG
JS91	TCACTTGTACAGCTCGTCCA
JS92	GCCGTTCTTCTGCTTGTCGGCGTGGTTCATGACCTTCTGTTT
JS93	CGCCCGGCCTGCAAGATC
JS96	ATGGCTTCTCCCCGTCCC
JS97	CAGGATCTTGCAGGCCGGGCGGGCGTCGCCAGATGCAGC
JS131	GATATCGACAACCTCTCCACTTAT
JS132	TACCAATGGGATCCCGTAATGGGGAGGAAGCAAGCCAAG
JS133	CCTTGGCTTGCTTCCTCCCCATTACGGGATCCCATTGGTAA
JS134	TAATAGGTCAAAGTCATGCCGCATCTTTGTTTGTATTATACTG
JS135	TATAATACAAACAAAGATGCGGCATGACTTTGACCTATTAG
JS136	GATATCGAAGCTTGACTTATTTGGT
JS148	AGCTCTGGAGGACCATGCG
JS150	CGCATGGTCCTCCAGAGCTGCCTTAGCCCCTTCAGGAAAGA
JS158	CGCATGGTCCTCCAGAGCTGACTTAGACCCTTCAGGAAAG
JS162	TACGCGGCGGGGCCCTCGATTTCTTTCCTGAAG
JS163	TACGCGGCGGGTCCCTCGATTTCTTTCCTGAAG
JS167	CCGCCGCGTATTGAGGGT
JS168	GCTCACCATACCACCGCTACCACCGGCGTCGCCAGATGCAG
JS169	TAATAGGTCAAAGTCATGCCTCAGGGGCAGGGCATGCT
JS170	AGCATGCCCTGCCCCTGAGGCATGACTTTGACCTATTA
JS171	CATATGCCATCTCGCACCCT
JS172	CATATGGTATAGTCTGGTCTTA
JS180	AGATCTCGACAATTTTCCGAGA
JS181	ACCCAACAACCATGATACCACAGTGCTGAGTGCTGGAGC
JS182	AGCTCCAGCACTCAGCACTGTGGTATCATGGTTGTTGGGT
JS183	CTGAACCGTACAATGAGATGAGCATCCACATGATCGACAG
JS184	TGTCGATCATGTGGATGCTCATCTCATTGTACGGTTCAGG
JS185	AGATCTTCGATTAAATTCTGCCA
JS201	CCTCGATTTCTTTCCTGAAGG
JS202	CTTCAGGAAAGAAATCGAGGGCCCCGCCGCGTATTGAG
JS203	CTTCAGGAAAGAAATCGAGGGACCCGCCGCGTATTGAG
JS204	CTCAATACGCGGCGGGGCCCTCGATTTCTTTCCTGAAGGGGCTAAGGCAGCTGATC
JS205	CCGCCGCGTATTGAGCGC
JS206	CTCAATACGCGGCGGGTCCCTCGATTTCTTTCCTGAAGGGTCTAAGTCAGCTGATC
JS219	GTTTTTCAGCAAGATCATATGCCATCTCGCACCCT
JS220	ATCTTCTAGAAAGATCATATGGTATAGTCTGGTCTTA
JS221	TCGCACAGCTGCAATACACAAAATTCTTACTGGGC
JS222	ATTGCAGCTGTGCGAGGTATGTCCCTGCGCAATTA
JS223	CAAAAGCACCGGTAGCGATAATGTGGTCTGCGCGGGCTTG
JS224	TATCGCTACCGGTGCTTTTGGTTTTTACGATGATACTTCG
JS225	CATCACCATCACCATCACTGAAATTCGCCTCAAACAATGCT
JS226	CTAATAGGTCAAAGTCATGCCCTGTCTGAGAGGAGGCACT
JS227	AGTGCCTCCTCTCAGACAGGGCATGACTTTGACCTATTAG
JS228	TCAGTGATGGTGATGGTGATGACCACCGCTACCACCGGCGTCGCCAGATGCAGC
ÖZG192	TCAGGGGCAGGGCATGCTCATGTAGAG
ÖZG207	GGTGGTAGCGGTGGTATGGTGAGC
ÖZG209	GGTGGTAGCGGTGGTAAGAGAAGATGGAAAAAGAATTTCATAG

### Protein isolation and western hybridization analyses

Proteins from *A*. *nidulans* were isolated using ground mycelia and extraction buffer B* (100 mM Tris-HCl pH 7.5, 300 mM NaCl, 10% glycerol, 2 mM EDTA pH 8.0, 0.02% NP-40, freshly supplemented with 2 mM DTT and Complete protease inhibitor cocktail (Roche)). For identification of phosphorylated DenA variants phosphatase inhibitor mixture (1 mM NaF, 0.5 mM Na_3_VO_4_, 8 mM β-glycerophosphat) was added. Protein concentration was determined by a modified Bradford assay [131]. Equal amounts of proteins were separated by 12% SDS-PAGE gels. Proteins were transferred onto nitrocellulose membrane (Whatman). In case of protein samples derived from asexual development Ponceau S (Sigma-Aldrich, 0.2% Ponceau S, 3% TCA) was used as reference for equally loaded protein amounts because the level of housekeeping proteins such as actin vary during development and Ponceau staining does not rely on a single protein for normalization or loading control [132]. Blocking was conducted using 5% milk powder (Sucofin) dissolved in TBS/T buffer (10 mM Tris-HCl pH 8.0, 150 mM NaCl, 0.05% Tween 20). Membranes were probed with primary antibodies such as Calmodulin-binding protein antibody (04–932, Millipore), phosphoserine/threonine antibody (ab17464, Abcam), α-GFP antibody (sc-9996, Santa Cruz), Tubulin antibody (T0926, Sigma-Aldrich), cullinA, cullinC or α-Nedd8 antibody (Genscript). As secondary antibodies horseradish peroxidase-coupled rabbit antibodies (G21234, Invitrogen) or mouse antibodies (115-035-003, Jackson ImmunoResearch) were used. Detection was performed using the Enhanced ChemiLuminescence method [133] The signal intensity was quantified with the Fusion-SL7 system and the Bio1D software (Peqlab).

### Co-purification methods

For Tandem affinity purification (TAP)-tag purification proteins extracted from ground mycelia were incubated with 400 μl IgG-agarose (GE Healthcare) for 2 h on a rotating platform at 4°C. The suspension was filtered with a PolyPrep column (Bio-Rad Laboratories). The remaining beads were washed twice with 10 ml IPP300 (25 mM Tris-HCl pH 8.0, 300 mM NaCl, 0.1% NP-40, 2 mM DTT), once with 10 ml IPP150 (25 mM Tris-HCl pH 8.0, 150 mM NaCl, 0.1% NP-40, 2 mM DTT), and once with 10 ml tobacco etch virus (TEV) cleavage buffer (25 ml Tris-HCl pH 8.0, 150 mM NaCl, 0.1% NP-40, 0.5 mM EDTA, 1 mM DTT). Columns were closed and 300 units of TEV protease in 1 ml of TEV cleavage buffer was added and incubated on a rotating platform overnight at 4°C. Cleaved proteins were eluted into a fresh PolyPrep column containing 300 μl calmodulin affinity resin (Agilent Technologies), equilibrated with 5 ml of calmodulin binding buffer (25 mM Tris-HCl pH 8.0, 150 mM NaCl, 1 mM Mg acetate, 1 mM imidazole, 2 mM CaCl_2_, 10 mM ß-mercaptoethanol). The elution step was repeated once with 1 ml of TEV cleavage buffer and 6 ml CBB and 6 μl of 1 M CaCl_2_, were added to the solution. The solution was incubated on a rotating platform for 2h at 4°C. The beads were washed twice with 1 ml of CBB containing 0.1% NP-40 and once with 1 ml of CBB containing 0.02% NP-40. Bound proteins were eluted three times with 1 ml of calmodulin elution buffer (25 mM Tris-HCl pH 8.0, 150 mM NaCl, 0.02% NP-40, 1 mM Mg-acetate, 1 mM imidazole, 20 mM EGTA, 10 mM ß-mercaptoethanol). The addition of trichloroacetic acid (TCA) to a concentration of 25% resulted in protein precipitation overnight at 4°C. The precipitate was collected by centrifugation with 16.000 rcf for 1 h at 4°C, washed with ice-cold acetone/0.05 M HCl and with acetone. Precipitated proteins were dried in a vacuum exhausted centrifuge. The proteins were resuspended in 30 μl 3x sample buffer and separated on SDS-PAGE followed either by protein staining, western hybridization or LC-MS/MS analysis.

For GFP-Trap purification 100–250 μl GFP-Trap beads (Chromotek) were washed twice with 2.5 ml pre-cooled B* buffer (100 mM Tris-HCl pH 7.5, 300 mM NaCl, 10% glycerol, 2 mM EDTA pH 8.0, 0.02% NP-40). Protein extracts and beads were mixed and incubated on a rotating platform at 4°C for 2h. The suspension was poured into a Bio-Rad PolyPrep column (Bio-Rad Laboratories), in which beads were washed twice with cold W300 buffer (10 mM Tris-HCl pH 7.5, 300 mM NaCl, 0.5 mM EDTA). Subsequently, the beads were washed twice with W500 buffer (10 mM Tris-HCl pH 7.5, 500 mM NaCl, 0.5 mM EDTA). Proteins bound to the beads either were dissociated by boiling at 95°C for 10 min or were eluted by adding 150 μl 0.2 M glycine pH 2.5 (incubation time: 30 sec under constant mixing) followed by neutralization with 9.5 μl 1 M Tris pH 10.4. This step was repeated three times. All buffers were freshly supplemented with 2 mM DTT and Complete protease inhibitor cocktail (Roche). For identification of DenA phosphorylation sites phosphatase inhibitor mixture (1 mM NaF, 0.5 mM Na_3_VO_4_, 8 mM β-glycerophosphat) was added. Samples were used for SDS-PAGE followed by western hybridization or LC-MS/MS analyses.

### Identification of proteins and phosphorylation sites by tandem mass spectrometry

Liquid chromatography coupled either to a LCQ DecaXP mass spectrometer or an Orbitrap Velos Pro™ Hybrid Ion Trap-Orbitrap mass spectrometer (Thermo Scientific GmbH) was employed for protein and phospho peptide identification, respectively. 1 to 6 μl of peptide containing sample solution were trapped and washed with 100% solvent A (98% water, 2% acetonitrile, 0.07% trifluoroacetic acid) on an Acclaim PepMap 100 pre-column (#164564, 100 μm x 2 cm, C18, 3 μm, 100 Å, Thermo Scientific GmbH) with a flow rate of 25 μl/min for 6 min. An Acclaim PepMap RSLC column (#164540, 75 μm or 50 cm, C18, 3 μm, 100 Å, Thermo Scientific GmbH) was used for analytical peptide separation by reverse phase chromatography. This was performed by typically applying a gradient from 98% solvent A (water, 0.1% formic acid) and 2% solvent B (80% acetonitrile, 20% water, 0.1% formic acid) to 42% solvent B within 95 min and to 65% solvent B within the next 26 min at a flow rate of 300 nl/min (solvents and acids from Thermo Scientific GmbH). On-line ionization of chromatographically eluting peptides was performed with the nanoelectrospray (nESI) using the Nanospray Flex Ion Source (Thermo Scientific GmbH) at 2.4 kV followed by a continuous transfer into the mass spectrometer. Full scans of the mass range of 300–1850 m/z were applied with the Orbitrap-FT analyzer at a resolution of 30,000 with parallel data-dependent top ten MS2 collision-induced dissociation (CID) fragmentation within the LTQ Velos Pro linear ion trap. CID fragmentation was used to analyse phospho peptide samples by applying the multistage activation (MSA) method as well as higher energy collisional dissociation (HCD) fragmentation in independent runs. When HCD fragmentation was performed, data-dependent top five MS2 fragmentation was used and fragment ions were analyzed in the orbitrap. The software XCalibur 2.2 was used for LC-MS method programming and data acquisition. The evaluation of phosphorylation site probabilities was performed by using phosphoRS and for identification of proteins MS/MS2 data were analyzed against the *A*. *nidulans* genome database [134] using the Sequest and Mascot search engine and the Proteome Discoverer Software version 1.4 [135,136]. Trypsin was used for sample digestion and a maximum of two missed cleavage sites was considered. For identification of DenA phosphorylation sites phosphopeptide enrichment with Ti0_2_ columns (Glygen Corporation) was performed prior to LC-MS/MS analysis. Carbamidomethyl at cysteines was set as fixed modification, whereas oxidation of methionines and phosphorylation of serines, threonines, and tyrosines were considered as variable modifications. Mass tolerances of precursors and fragment ions were 10 ppm and 0.6 Da, respectively. False discovery rates were calculated with the Proteome Discoverer using the revert-decoy mode. The filter for valid peptide sequence matches was set to 0.01.

### Phos-tag acrylamide

For determining the phosphorylation status of DenA the Phos-tag based Mobility Shift Detection of Phosphorylated Proteins (Wako) was used. All solutions required were prepared according to manufacturer’s instructions. 100 μl of Phos-tag AAL-107 dissolved in methanol and water was directly added to SDS gels. After electrophoresis the proteins were fixed in the gel by soaking in fixation solution (50% H_2_O, 40% v/v methanol, 10% v/v acetic acid) for 10 min with gentle agitation. Proteins were stained using Coomassie colloidal blue solution for 2h and washed in destaining solution.

### Quantification methods

Radial growth tests were performed by point-inoculation of 5000 spores. Plates were incubated at 37°C for six days and colony diameter was measured each day. Quantification of conidiospores was performed by point-inoculation of 5000 spores. Plates were incubated at 37°C for four days and distinct regions of the colony were excised with the end of a 0.2 ml tip, vortexed for 30 min in 0.5 ml of saline and spores were counted. Membranes, including septa, were stained with 1 mM FM4-64 (Invitrogen) to quantify septa within vegetative hyphae. The compartment volume was calculated by using the formula: V = h π r^2^. The width of the hypha was calculated as diameter (d) and the distance between septa as height (h), respectively. Pixel density values for quantification of the western hybridization signals were obtained as described previously [16].

### Microscopy

*A*. *nidulans* colonies were visualized with an Olympus CS30 digital camera combined with an Olympus SZX-ILLB2-200 binocular. Pictures were processed with the cellSens software (Olympus). For fluorescence microscopy 500–2000 spores were inoculated in an 8-well borosilicate coverglass system (Thermo Scientific) containing 400 μl liquid minimal medium. Microscopy was performed with a Zeiss Axio Observer Z.1 system with Zeiss PlanAPOCHROMAT 63x/1.4oil. Pictures and movies were obtained with a QuantEM:512SC (Photometrics) or a Coolsnap HQ^2^ (Photometrics) camera and the SlideBook 5.0 imaging software (Intelligent Imaging Innovations Inc.). Membranes were stained with 1 mM FM4-64 (Invitrogen) and mitochondria with 50 nM MitoTracker Red CMXRos (Invitrogen). Nuclei were visualized with ectopically integrated *h2A*::*rfp*. Exposure times for GFP: 1000 ms, RFP: 100 ms, DIC: 100 ms and in BiFC experiments exposure times were YFP: 1000 ms, RFP: 100 ms, DIC: 100 ms.

### Computational methods

BLAST searches were conducted at the National Center for Biotechnology Information [137]. Gene and protein information was provided by CADRE [138] or AspGD [134]. DNA sequence analyses were carried out using the Lasergene 8.0 software (Dnastar). Multiple sequence alignment was performed using MultAlign [56].

## Supporting Information

S1 Fig Influence of deneddylases on cullin deneddylation. Western hybridization of wild type (WT) and deneddylase deficient strains. Protein crude extracts from vegetative grown mycelia of WT, Δ*csnE*, Δ*denA* and *csnE*/*denA* double deletion strains were compared. **(A)** CullinA versions were visualized using cullinA antibody. **(B)** CullinC antibodies were used to detect cullinC variants. Detected bands represent unneddylated cullins (CulA/CulC), mononeddylated cullins (CulA-N8/CulC-N8) and hyperneddylated cullins (marked with asterisks). The ratio of neddylated CulA and CulC to the respective deneddylated protein versions was calculated (lower panels). Three independent experiments were used to generate these data. The mean values with standard deviations are shown.
(TIF)

S2 Fig Purified DenA-GFP and overexpressed GFP by GFP-Trap. Colloidal blue stained SDS gels and western hybridization applying GFP antibody on **(A)** DenA-GFP or **(B)** overexpressed GFP (OE *gfp*). Respective proteins were enriched by GFP-Trap during vegetative (left) and asexual (right) conditions. FT = flow through; WI/WII = washing steps; EI/EII/EIII = elution steps.
(TIF)

S3 Fig Identification of DenA phosphorylation sites by LC-MS/MS. **(A)** DenA amino acid sequence with peptides (highlighted in green) and respective phosphorylation sites (P) identified by LC-MS/MS. **(B)** Identified phosphorylation sites of DenA with phospho site probability (pRS, [139]). A3 = vegetative DenA, Sequest; B3 = asexual DenA, Sequest; A5 = vegetative DenA, Mascot; B5 = asexual DenA, Mascot. Peptide scores for search engine Sequest and Mascot indicated by XCorr and IonScore, respectively.
(TIF)

S4 Fig Multiple alignment of DenA C-terminus with *Aspergilli* species and higher eukaryotes. Sequences were aligned from the deduced DenA C-termini of *Aspergillus nidulans*, *Aspergillus clavatus*, *Aspergillus fumigatus Af293*, *Aspergillus terreus*, *Arabidopsis thaliana*, *Mus musculus*, *Homo sapiens* and *Caenorhabditis elegans*. High consensus value (80%) = red, low consensus value (50%) = blue [56]. Identified phosphorylation sites of *A*. *nidulans* are marked with asterisks.
(TIF)

S5 Fig Phenotypical characterization of strains with mutated DenA variants. DenA control strain (*denA*), *denA* deletion strain (Δ*denA*), complementation strain (Δ*denA* +*denA*), *denA* under control of an inducible promotor (*niiA*::*denA*) and respective DenA variants with mutated phosphorylation sites (alanine (A) mimics unphosphorylated and aspartate (D) constant phosphorylated versions at these positions) in *denA* deletion background were analyzed. **(A)** The phenotype of the DenA variant carrying alanines instead of the phosphorylated serine residues is reminiscent of *denA* deletion phenotype. **(B)** Equal amount of spores were incubated during asexual development inducing conditions under limited pyrimidine supply. Quantification of conidiospores was performed in triplicates and the mean values with standard deviations are shown.
(TIF)

S6 Fig Protein localization and occurrence of DipA. **(A & B)** Western hybridizations of DipA-GFP and DenA-GFP. Samples were taken from vegetative cultures and during different time points (in hours) of illumination induced asexual development. SDS gels were loaded with equal amounts of protein crude extracts. Membranes were treated with GFP-antibody and as loading control staining with Ponceau was applied. Protein weight of DipA-GFP was 99.5 kDa, DenA-GFP was 54.5 kDa and free GFP was 25 kDa. **(C)** Localization of DipA-GFP was followed by fluorescence microscopy. Several distinct spots were visible in the cytoplasm and at septa. Nuclei are marked with N and septa with S. Nuclei were visualized with expressed *rfp*::*h2A*. As control wild type hyphae were used. Scale bar: 5 μm. **(D)** Time lapse observations over 190 seconds. Shuttling of DipA-GFP is highlighted and followed by white arrows.
(TIF)

S7 Fig Localization of DenA in *dipA* deletion strain. The overall cellular distribution of DenA-GFP was not affected by the absence of DipA. DenA-GFP in *dipA* deletion strain occurred inside nuclei (N), in the cytoplasm and predominantly at septa (S). As control wild type hyphae were used. Nuclei were visualized with expressed *rfp*::*h2A* and membranes were stained with FM4-64. Scale bar: 5 μm.
(TIF)

S1 Video Transport of DenA-DipA interaction complex analyzed by Bimolecular fluorescence complementation (BiFC) studies. Nuclei of *A*. *nidulans* hyphae were visualized with RFP-H2A fusion and membranes were stained with FM4-64. The video consists of 17 frames with an average time lapse interval of 10 s and a setting of 4 frames/s. Finale frame duration 0.2 s. DIC: 100 ms, YFP: 1000 ms, RFP: 100 ms. Scale bar: 5μm.
(MOV)

S2 Video Transport of DenA-DipA interaction complex analyzed by Bimolecular fluorescence complementation (BiFC) studies. Nuclei of *A*. *nidulans* hyphae were visualized with RFP-H2A fusion. The video consists of 72 frames with an average time lapse interval of 3.2 s and a setting of 10 frames/s. Finale frame duration 0.1 s. DIC: 100 ms, YFP: 1000 ms, RFP: 100 ms. Scale bar: 5μm.
(MOV)

S3 Video Transport of DenA-DipA interaction complex analyzed by Bimolecular fluorescence complementation (BiFC) studies. Mitochondria of *A*. *nidulans* hyphae were visualized with MitoTracker. The video consists of 35 frames with an average time lapse interval of 3.1 s and a setting of 10 frames/s. Finale frame duration 0.1 s. DIC: 100 ms, YFP: 1000 ms, RFP: 100 ms. Scale bar: 5μm.
(MOV)
