# Supplementary figures and images for: The DenA/DEN1 Interacting Phosphatase DipA Controls Septa Positioning and Phosphorylation-Dependent Stability of Cytoplasmatic DenA/DEN1 during Fungal Development
Source: PLoS Genet. 2016 Mar 24;12(3):e1005949. doi: 10.1371/journal.pgen.1005949 (PMC4806917; doi:10.1371/journal.pgen.1005949)

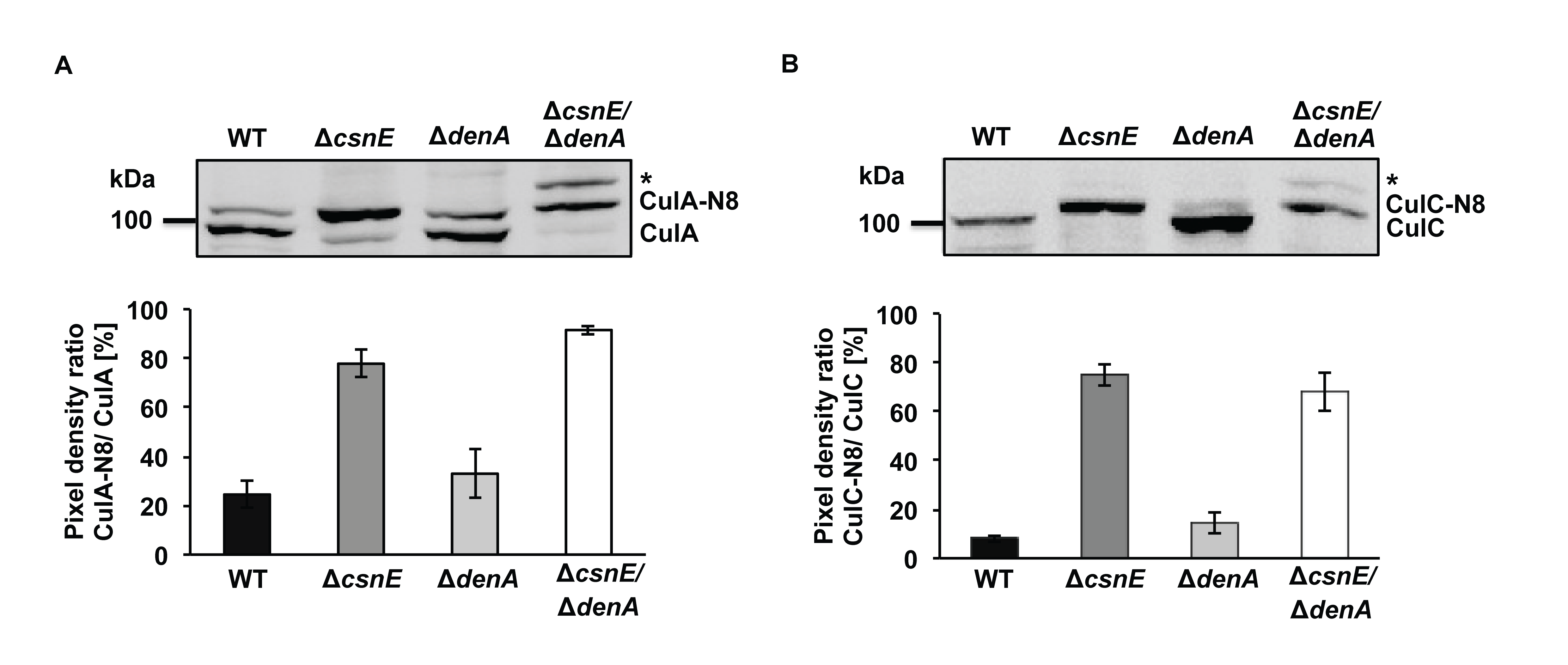

Supplement: S1 Fig — Western hybridization of wild type (WT) and deneddylase deficient strains. Protein crude extracts from vegetative grown mycelia of WT, ΔcsnE, ΔdenA and csnE/denA double deletion strains were compared. (A) CullinA versions were visualized using cullinA antibody. (B) CullinC antibodies were used to detect cullinC variants. Detected bands represent unneddylated cullins (CulA/CulC), mononeddylated cullins (CulA-N8/CulC-N8) and hyperneddylated cullins (marked with asterisks). The ratio of neddylated CulA and CulC to the respective deneddylated protein versions was calculated (lower panels). Three independent experiments were used to generate these data. The mean values with standard deviations are shown. (TIF) [file pgen.1005949.s001.tif]

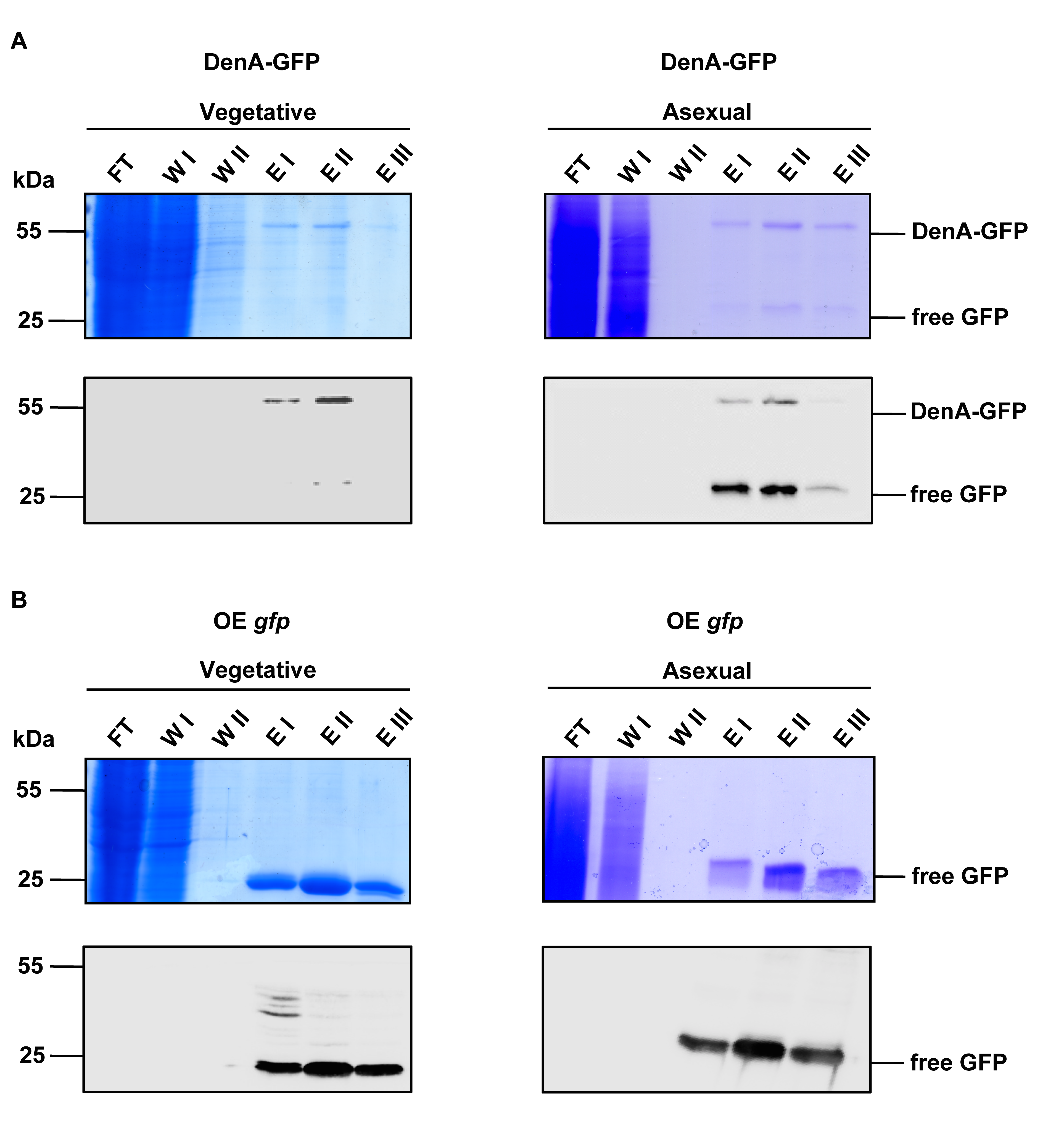

Supplement: S2 Fig — Colloidal blue stained SDS gels and western hybridization applying GFP antibody on (A) DenA-GFP or (B) overexpressed GFP (OE gfp). Respective proteins were enriched by GFP-Trap during vegetative (left) and asexual (right) conditions. FT = flow through; WI/WII = washing steps; EI/EII/EIII = elution steps. (TIF) [file pgen.1005949.s002.tif]

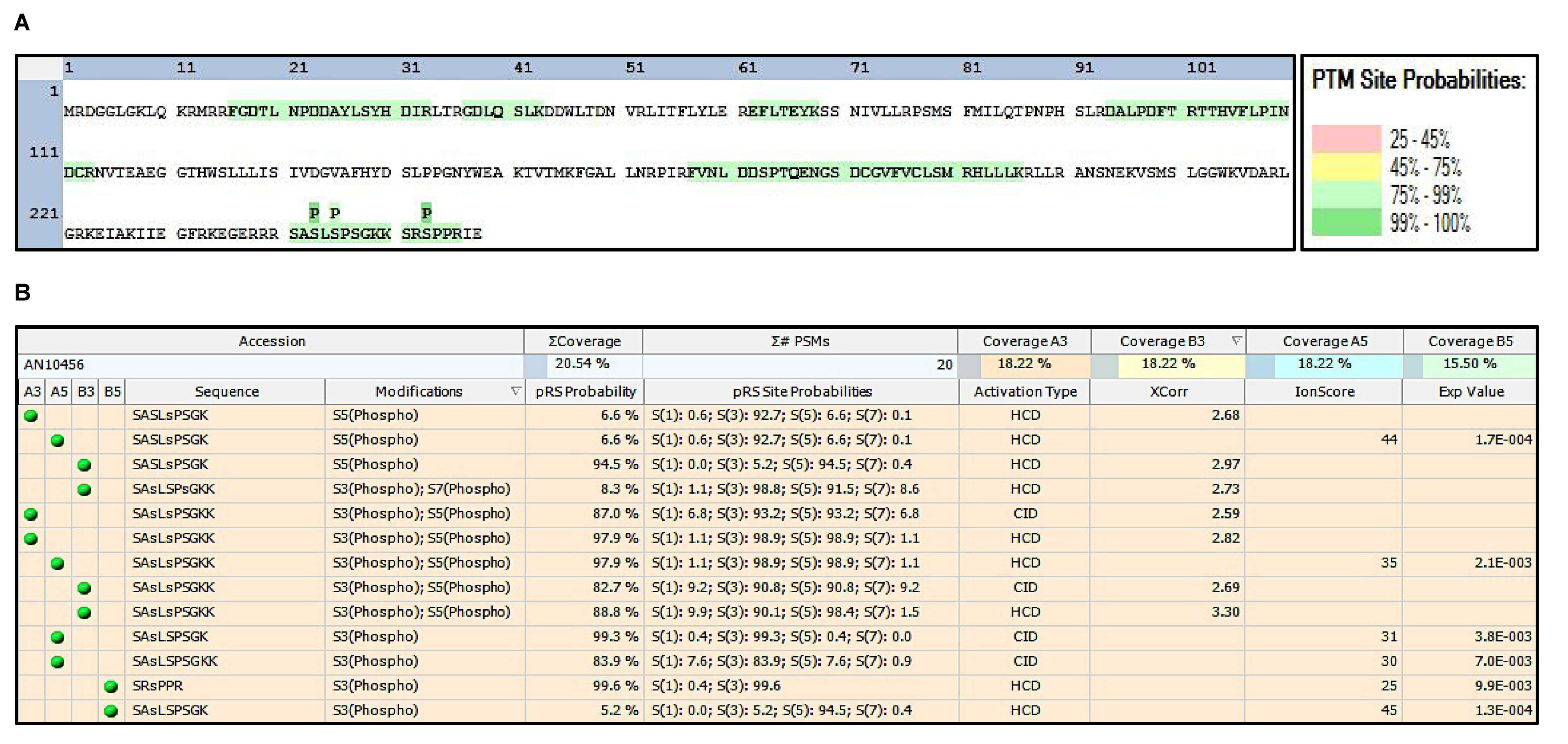

Supplement: S3 Fig — (A) DenA amino acid sequence with peptides (highlighted in green) and respective phosphorylation sites (P) identified by LC-MS/MS. (B) Identified phosphorylation sites of DenA with phospho site probability (pRS, [139]). A3 = vegetative DenA, Sequest; B3 = asexual DenA, Sequest; A5 = vegetative DenA, Mascot; B5 = asexual DenA, Mascot. Peptide scores for search engine Sequest and Mascot indicated by XCorr and IonScore, respectively. (TIF) [file pgen.1005949.s003.tif]

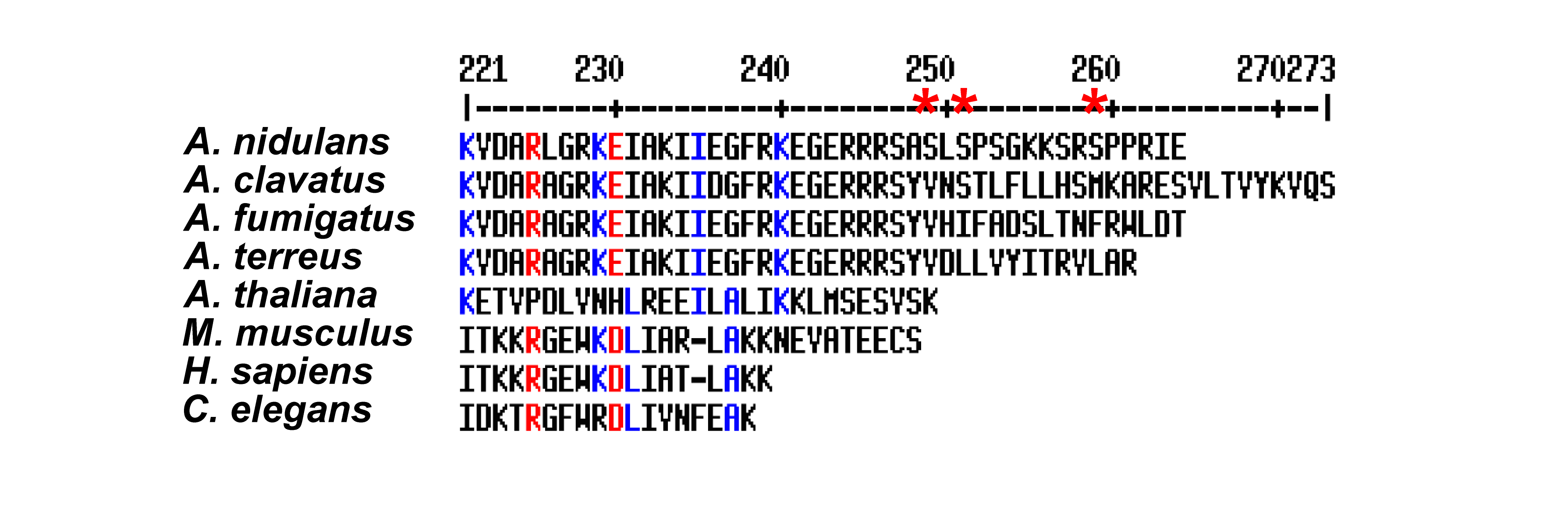

Supplement: S4 Fig — Sequences were aligned from the deduced DenA C-termini of Aspergillus nidulans, Aspergillus clavatus, Aspergillus fumigatus Af293, Aspergillus terreus, Arabidopsis thaliana, Mus musculus, Homo sapiens and Caenorhabditis elegans. High consensus value (80%) = red, low consensus value (50%) = blue [56]. Identified phosphorylation sites of A. nidulans are marked with asterisks. (TIF) [file pgen.1005949.s004.tif]

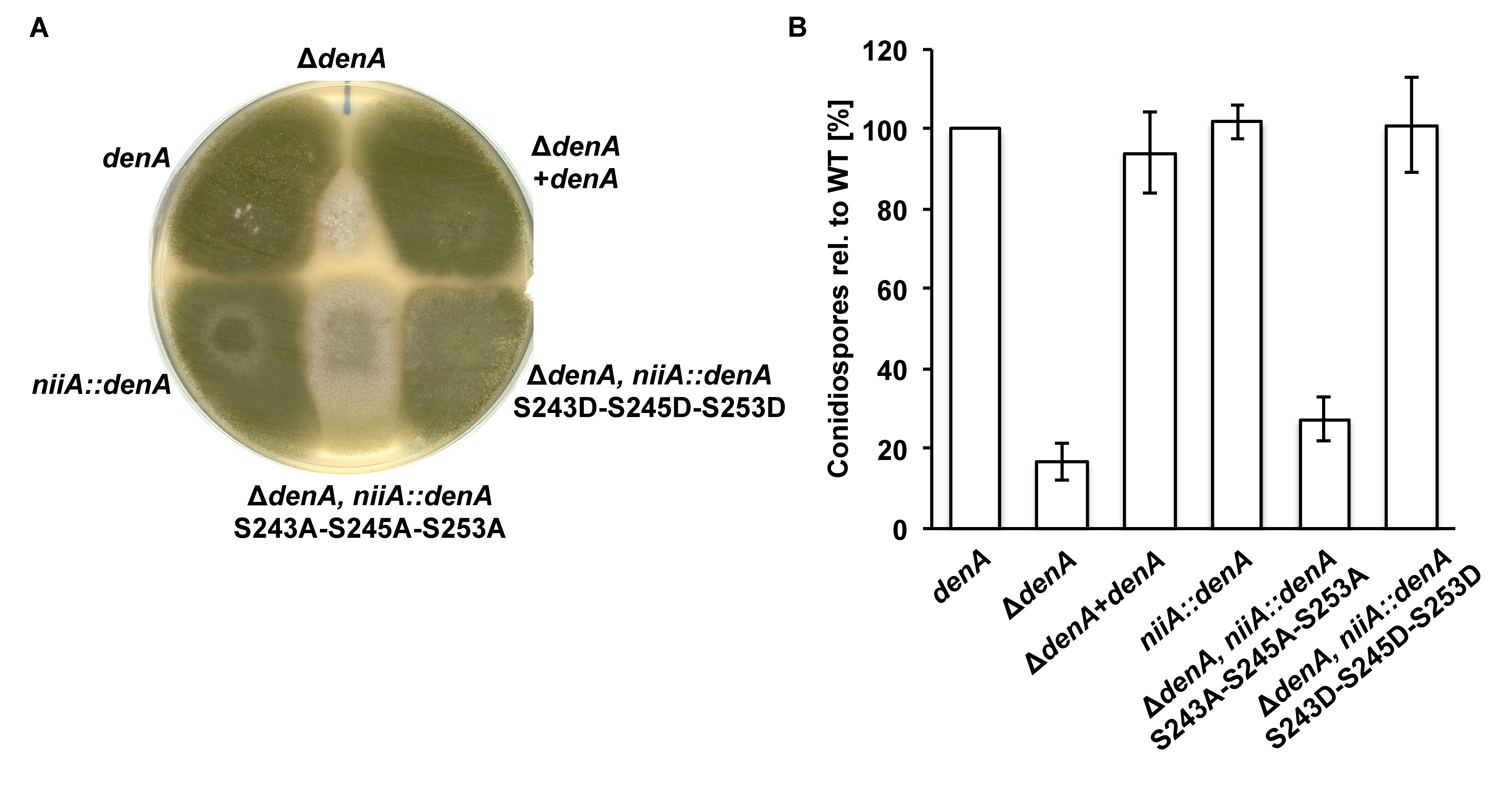

Supplement: S5 Fig — DenA control strain (denA), denA deletion strain (ΔdenA), complementation strain (ΔdenA +denA), denA under control of an inducible promotor (niiA::denA) and respective DenA variants with mutated phosphorylation sites (alanine (A) mimics unphosphorylated and aspartate (D) constant phosphorylated versions at these positions) in denA deletion background were analyzed. (A) The phenotype of the DenA variant carrying alanines instead of the phosphorylated serine residues is reminiscent of denA deletion phenotype. (B) Equal amount of spores were incubated during asexual development inducing conditions under limited pyrimidine supply. Quantification of conidiospores was performed in triplicates and the mean values with standard deviations are shown. (TIF) [file pgen.1005949.s005.tif]

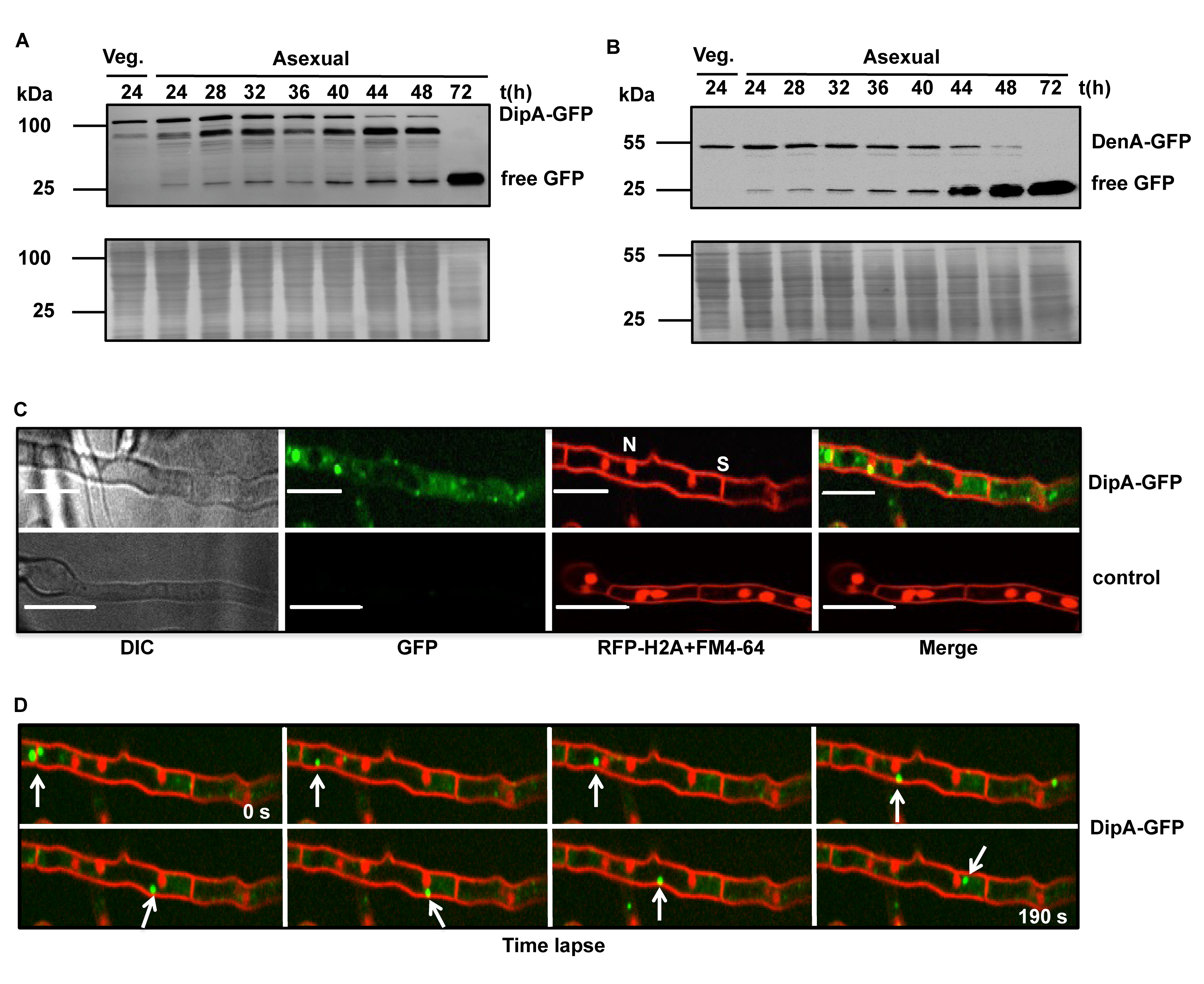

Supplement: S6 Fig — (A & B) Western hybridizations of DipA-GFP and DenA-GFP. Samples were taken from vegetative cultures and during different time points (in hours) of illumination induced asexual development. SDS gels were loaded with equal amounts of protein crude extracts. Membranes were treated with GFP-antibody and as loading control staining with Ponceau was applied. Protein weight of DipA-GFP was 99.5 kDa, DenA-GFP was 54.5 kDa and free GFP was 25 kDa. (C) Localization of DipA-GFP was followed by fluorescence microscopy. Several distinct spots were visible in the cytoplasm and at septa. Nuclei are marked with N and septa with S. Nuclei were visualized with expressed rfp::h2A. As control wild type hyphae were used. Scale bar: 5 μm. (D) Time lapse observations over 190 seconds. Shuttling of DipA-GFP is highlighted and followed by white arrows. (TIF) [file pgen.1005949.s006.tif]

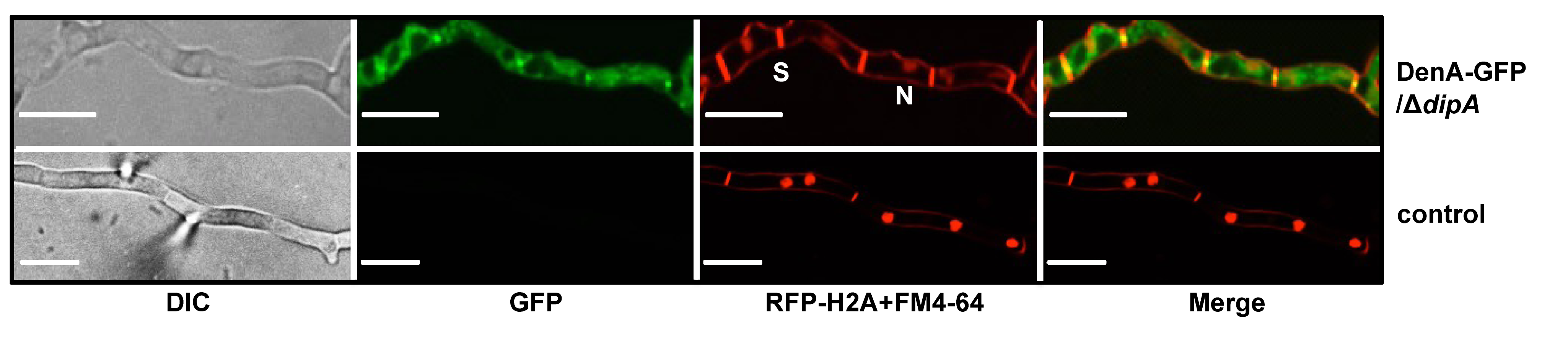

Supplement: S7 Fig — The overall cellular distribution of DenA-GFP was not affected by the absence of DipA. DenA-GFP in dipA deletion strain occurred inside nuclei (N), in the cytoplasm and predominantly at septa (S). As control wild type hyphae were used. Nuclei were visualized with expressed rfp::h2A and membranes were stained with FM4-64. Scale bar: 5 μm. (TIF) [file pgen.1005949.s007.tif]
